# Effects of different potassium fertilization rates on root-soil interaction in Korla fragrant pear orchards

**DOI:** 10.3389/fpls.2026.1899783

**Published:** 2026-10-07

**Authors:** Kailong Wang, Yuan Hu, Xiaolei Sun, Pengxue Jia, Enduo Liu, Xintong Gao, Zhongping Chai

**Affiliations:** 1 College of Resources and Environment, Xinjiang Agricultural University, Urumqi, China; 2 Xinjiang Key Laboratory of Soil and Plant Ecological Processes, Xinjiang Agricultural University, Urumqi, China

**Keywords:** enzyme activity, Korla fragrant pear, potassium fertilization, root anatomical structure, root vitality, soil physicochemical properties

## Abstract

**Introduction:**

To clarify the responses of the soil–root system in Korla fragrant pear orchards to different potassium application rates and their relationships with yield formation, this study evaluated the effects of potassium fertilization across the vertical soil profile, with the aim of providing a basis for rational potassium management in drip-irrigated Korla fragrant pear orchards.

**Methods:**

A field experiment was conducted in a 7–8-year-old Korla fragrant pear orchard under drip irrigation. Four potassium treatments were established: K0 (0 kg hm^-2^), K1 (75 kg hm^-2^), K2 (150 kg hm^-2^), and K3 (225 kg hm^-2^). Soil physicochemical properties, soil enzyme activities, root vitality, and fruit yield were measured at depths of 0–20, 20–40, and 40–60 cm. Mantel tests, principal component analysis (PCA), and partial least squares structural equation modeling (PLS-SEM) were used to analyze relationships among the measured variables.

**Results:**

Potassium application generally improved soil nutrient availability and the activities of several soil enzymes while reducing soil pH and electrical conductivity (EC), although the magnitude of these responses varied with potassium application rate and soil depth. Potassium application significantly increased root vitality and was accompanied by adjustments in root anatomical traits, including cortical thickness, vascular tissue cell composition, vessel area, and root cross-sectional area. Most soil, enzyme activity, and root-related indicators showed favorable responses under the K2 treatment, and fruit yield under K2 was 47.36% higher than that under K0. PCA and PLS-SEM indicated strong associations among soil physicochemical properties, enzyme activities, root traits, and fruit yield.

**Discussion:**

Under the soil conditions, tree age, and drip-irrigation management regime of this study, the 150 kg hm^-2^ potassium treatment showed favorable integrated soil–root responses and yield performance. However, its broader suitability requires further validation through multi-year and multi-site experiments.

## Graphical Abstract

**Figure:**
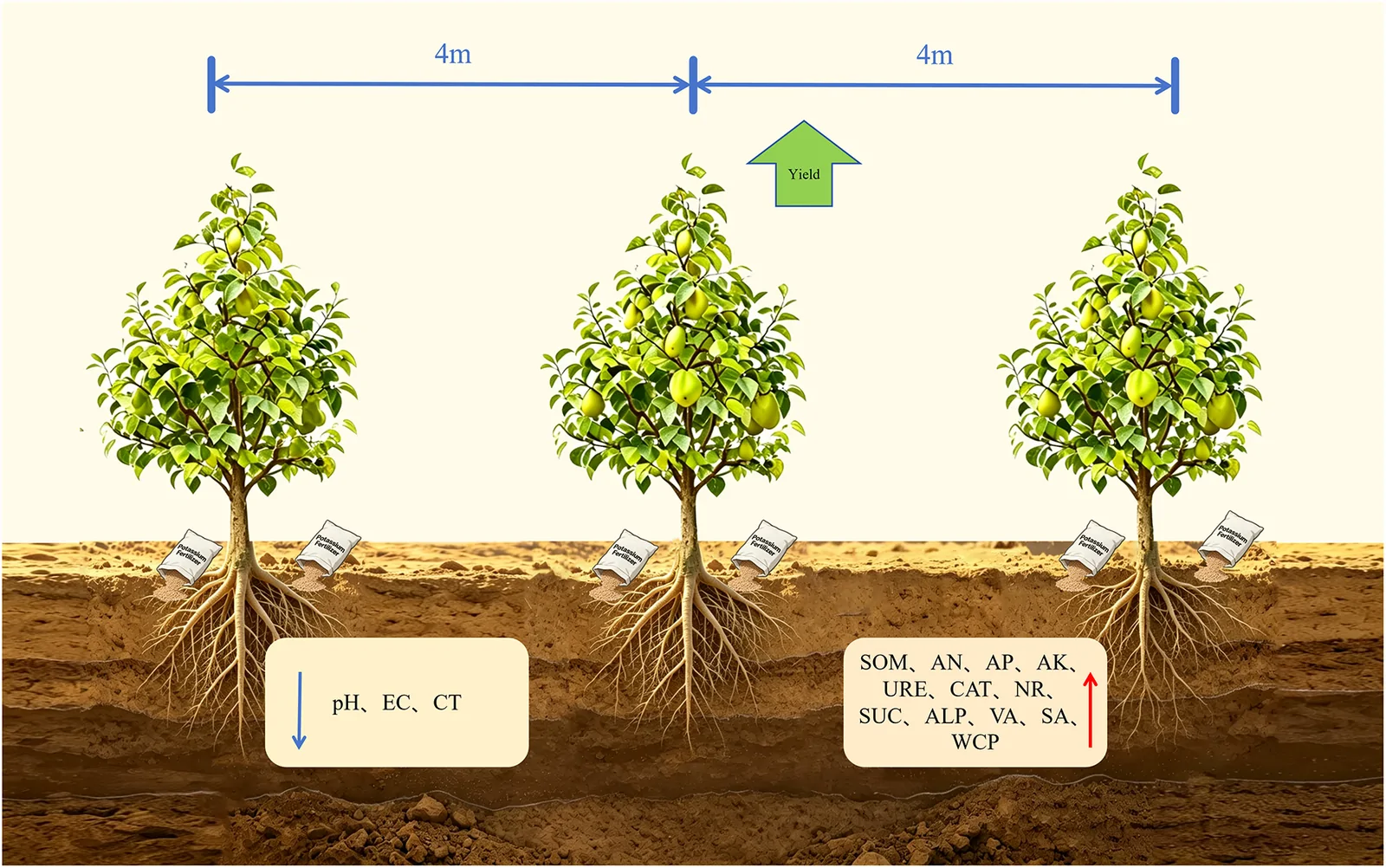

## 1 Introduction

Potassium (K) is the most abundant inorganic cation in plants and participates in a wide range of physiological processes, including photosynthesis, enzymatic reactions, osmotic regulation, and assimilate transport, thereby playing an important role in crop yield formation and quality improvement (Huai et al., 2022). Numerous studies have shown that appropriate potassium fertilization can increase plant K uptake (Mardanluo et al., 2018; Wu et al., 2019), enhance stress resistance (KS et al., 2021; Li and Si, 2019; Shahid et al., 2019), and promote plant growth and development (Shahid et al., 2020). However, both insufficient and excessive K supply can disrupt physiological metabolism and nutrient balance in plants, thereby limiting yield and quality formation (Tewari et al., 2021). Soil and fertilizers are the main sources of K available to plants (Zörb et al., 2014). With increasing crop yield levels and cropping intensity, together with imbalanced nutrient inputs in agricultural production, soil K reserves have continued to decline, resulting in an overall K deficit (Philp et al., 2021). China has relatively limited potash resources, and more than 60% of the potassium fertilizer used in agricultural production depends on imports (Hao et al., 2025). Therefore, improving potassium use efficiency and determining appropriate K application rates are important for maintaining stable crop production and promoting green agricultural development. In perennial fruit trees, K is involved not only in vegetative growth and fruit development but is also closely associated with root development and orchard soil physicochemical properties (He et al., 2025; Pourranjbari Saghaiesh and Souri, 2018). Korla fragrant pear (*Pyrus sinkiangensis* Yü) is a high-quality specialty pear cultivar in Xinjiang and is highly responsive to K supply. However, practical production is still characterized by relatively high nitrogen inputs and insufficient K supply (**Figure 1**) (Yan et al., 2026), making rational regulation of K fertilizer inputs an important component of nutrient management in Korla fragrant pear orchards.

**Figure 1:**
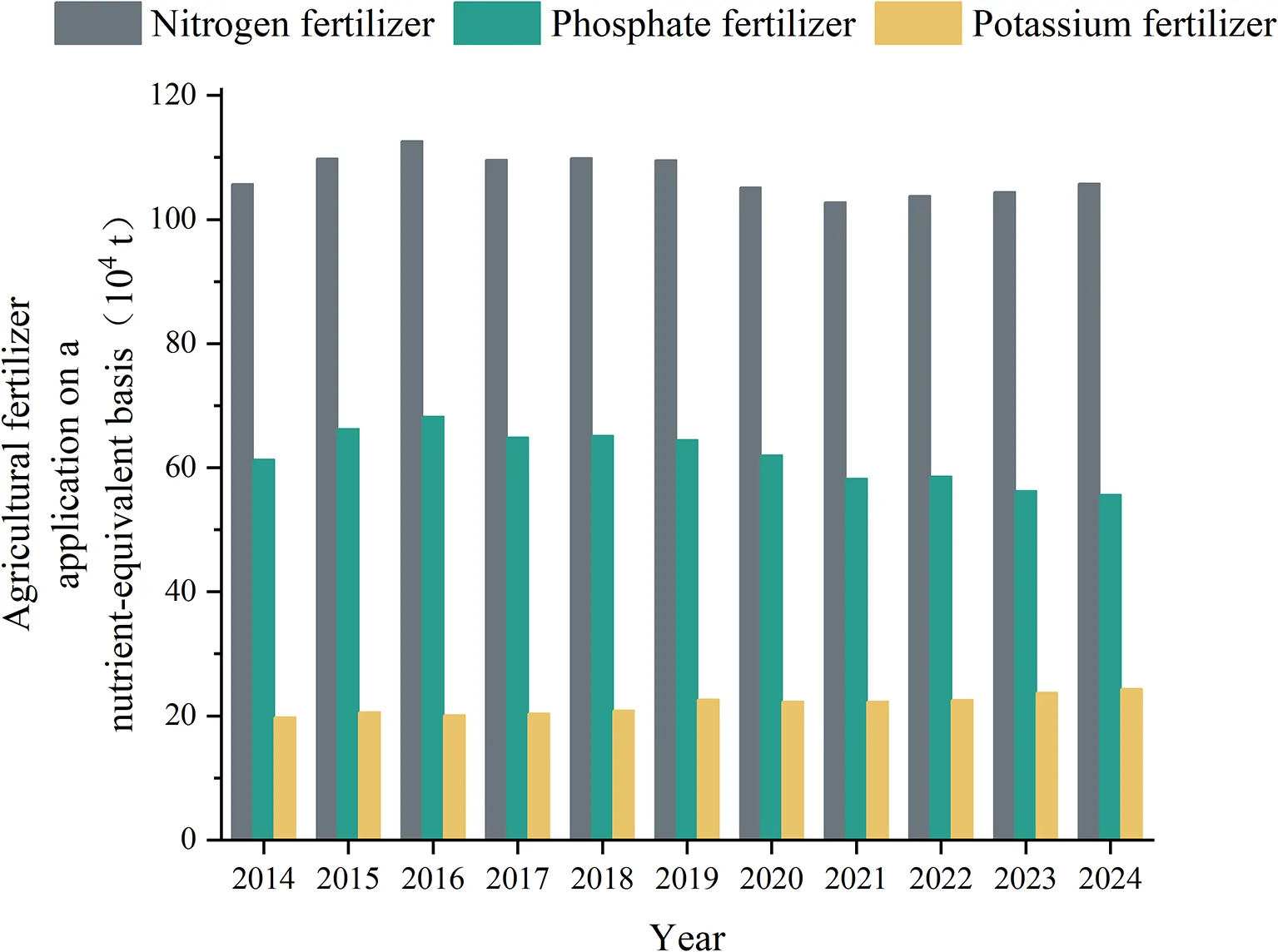
The National Bureau of Statistics of Xinjiang in 2014–2024 years of agricultural fertilizer application amount of pure statistics.

In recent years, research on K nutrition in pear trees has gradually expanded from a primary focus on yield responses to belowground ecological processes. Chen et al. (2021) found marked differences in soil nutrient status among different soil layers in pear orchards, while Sun et al. (2023) conducted a comprehensive survey of mineral nutrients in soils at different depths, as well as in leaves and fruits, across representative pear orchards in the Bohai Bay and Loess Plateau regions of China. Taken together, these findings indicate that nutrient assessment in perennial fruit trees should account for spatial heterogeneity along the soil profile. Li et al. (2025) reported that absorptive roots of Korla fragrant pear were mainly distributed within the 0–60 cm soil layer, with the 0–40 cm layer representing an important overlap zone between nutrient enrichment and root activity. Meanwhile, soil organic matter, alkali-hydrolyzable nitrogen, available phosphorus, and available potassium showed pronounced spatial heterogeneity in both vertical and horizontal directions and were jointly influenced by fertilizer placement, water movement, and root system architecture (Han et al., 2023; Meng et al., 2023; Wang et al., 2024; Xie et al., 2025; Yu et al., 2025). Yang et al. (2025) further showed that different K application rates altered soil nutrient status, microbial community composition, and functional genes in Korla fragrant pear orchards and also affected fruit yield, suggesting that K fertilization may influence tree productivity by modifying belowground ecological processes. As the key interface linking the soil environment and tree growth, roots can plastically adjust their physiological activity and anatomical structure in response to external resource conditions. Li et al (Li et al., 2023)) demonstrated that the anatomical characteristics of the cortex and vascular tissues in fine roots are closely related to resource acquisition and transport functions, reflecting plant adaptive strategies to resource environments. However, existing studies on K fertilization in Korla fragrant pear have mainly focused on individual components such as soil nutrients, microbial communities, or yield. Systematic information remains limited regarding the coordinated responses among soil physicochemical properties, enzyme-mediated nutrient transformation, root physiological activity, and root anatomical structure across different soil layers. In particular, it remains unclear how these vertically stratified root–soil responses are associated with changes in fruit yield. Therefore, compared with previous local K fertilization studies that mainly focused on soil nutrients, microbial communities, and yield responses, the present study further integrated soil physicochemical properties, enzyme activities, and root vitality within a common vertical soil-profile analytical framework.

Accordingly, this study was conducted in a 7–8-year-old Korla fragrant pear orchard under drip irrigation, with four K treatments: K0 (0 kg hm^-2^), K1 (75 kg hm^-2^), K2 (150 kg hm^-2^), and K3 (225 kg hm^-2^). The effects of different K application rates on soil physicochemical properties, enzyme activities, root vitality, and fruit yield were evaluated at soil depths of 0–20, 20–40, and 40–60 cm. Mantel tests, principal component analysis (PCA), and partial least squares structural equation modeling (PLS-SEM) were further used to examine the relationships among the soil environment, root function, and fruit yield under different K application rates. We hypothesized that: (1) moderate K application would improve soil nutrient availability and enhance the activities of related soil enzymes, whereas further increases in K application rate might weaken the positive responses of some soil nutrients and enzyme activities; (2) K-induced changes in the soil environment would be associated with increased root vitality and adjustments in root anatomical structure, and these responses would differ among soil layers because of the vertical heterogeneity of the root-zone soil environment; and (3) K application rate would be continuously associated with soil physicochemical properties, enzyme activities, root traits, and fruit yield, with the integrated response potentially following a pathway of “soil physicochemical properties–soil enzyme activities–root traits–yield.” The objective of this study was to characterize the coordinated responses of the root–soil system to different K application rates at the soil-profile scale and to provide a theoretical basis for rational K fertilization in drip-irrigated Korla fragrant pear orchards in arid regions.

## 2 Materials and methods

### 2.1 Experimental site description

The experiment was conducted in Awati Township, Korla City, Xinjiang Uygur Autonomous Region (86°4′E, 41°41′N). The study area is located on the southern foothills of the Tianshan Mountains, at the northeastern edge of the Tarim Basin, adjacent to the northern edge of the Taklamakan Desert, and is characterized by a warm temperate continental arid climate. The region receives approximately 2,800–3,100 hours of annual sunshine, with a mean annual temperature of 11.0–12.0 °C, and an average annual precipitation of about 58 mm. Evaporation is extremely high, ranging from 2,700 to 3,000 mm, with total solar radiation of 5,600–6,400 mJ·cm^-2^, effective accumulated temperature of 4,200–4,500 °C, and a frost-free period of 210–240 days. Prior to the experiment, the main physicochemical properties of the orchard soil were as follows: SOM 18.46 g·kg^-1^, TN 0.91 g·kg^-1^, AN 36.09 mg·kg^-1^, AP 59.67 mg·kg^-1^, AK 186.67 mg·kg^-1^, and pH 7.75.

### 2.2 Experimental design

The experiment was conducted in a moderately fertile orchard of 7–8-year-old Korla fragrant pear (*Pyrus sinkiangensis* Yü) trees under drip irrigation to evaluate the effects of different potassium application rates. Under a uniform irrigation quota of 7,800 m^3^·hm^-2^, four potassium treatments were established: K0 (0 kg·hm^-2^), K1 (75 kg·hm^-2^), K2 (150 kg·hm^-2^), and K3 (225 kg·hm^-2^), with three independent replicates per treatment. The experiment followed a randomized complete block design (RCBD), with the three replicates spatially arranged as three blocks along the direction of the drip-irrigation lateral lines to minimize systematic errors caused by soil heterogeneity. Each block contained all four potassium treatments, and treatment positions within each block were independently randomized rather than arranged sequentially. Each replicate was treated as an independent experimental plot, resulting in a total of 12 plots. Each plot consisted of five consecutive Korla fragrant pear trees with relatively uniform growth conditions within the same row. Thus, each treatment included 15 experimental trees, and the entire experiment comprised 60 trees. The pear trees were grafted onto Pyrus betulifolia rootstock, with a plant spacing of 1.5 m × 4 m (**Figure 2A**) and a planting density of 1,500 trees·hm^-2^. Based on the plant spacing, the net area of each plot was approximately 30 m^2^. To reduce water and nutrient movement between adjacent treatments and minimize edge effects, multiple buffer measures were established between plots. Two continuous rows of Korla fragrant pear trees were maintained between adjacent experimental plots as inter-row buffer zones. In addition, one buffer tree was placed at each end of every experimental row to minimize row-end edge effects, and two outer buffer rows were established around the entire experimental area. All buffer trees received the same irrigation quota of 7,800 m^3^·hm^-2^ but no experimental potassium fertilizer, equivalent to the K0 treatment, and were otherwise managed according to conventional orchard practices. Adjacent blocks were separated longitudinally by at least 4 m, corresponding to one row spacing, to minimize interference among blocks. Detailed irrigation and potassium fertilization practices are presented in **Table 1**. Semi-decomposed sheep manure from locally housed sheep, containing 18.52% total carbon, 0.76% total nitrogen, 0.52% total phosphorus, and 0.45% total potassium, was applied once as a basal fertilizer at 27,000 kg·hm^-2^ after fruit harvest in the previous autumn by surface broadcasting followed by soil incorporation. Nitrogen, phosphorus, and potassium fertilizers were applied during the current growing season. For nitrogen fertilizer, 40% of the total urea rate (46% N) was applied as basal fertilizer before bud break, and the remaining 60% was applied as topdressing at different growth stages. Triple superphosphate (46% P_2_O_5_) was applied once as a basal fertilizer before bud break. For potassium fertilizer, 40% of the total potassium sulfate rate (51% K_2_O) was applied as basal fertilizer before bud break, except in the K0 treatment, and the remaining 60% was applied as topdressing at different growth stages. Basal nitrogen, phosphorus, and potassium fertilizers were applied in circular trenches (**Figure 2B**). The fertilizer trenches were located 50–80 cm from the trunk beneath the canopy, with a width of 30 cm and a depth of 30–60 cm. Topdressing was applied through the drip-irrigation system using a differential-pressure fertilizer tank with a capacity of 15 L. Fertilizers were completely dissolved one day before application. Fertigation followed a “1/4–1/2–1/4” irrigation schedule: clear water was applied during the first quarter of the irrigation period, fertilizer solution was applied during the middle half, and clear water was applied again during the final quarter to flush the irrigation lines. All other field management practices were consistent with local orchard management practices.

**Figure 2:**
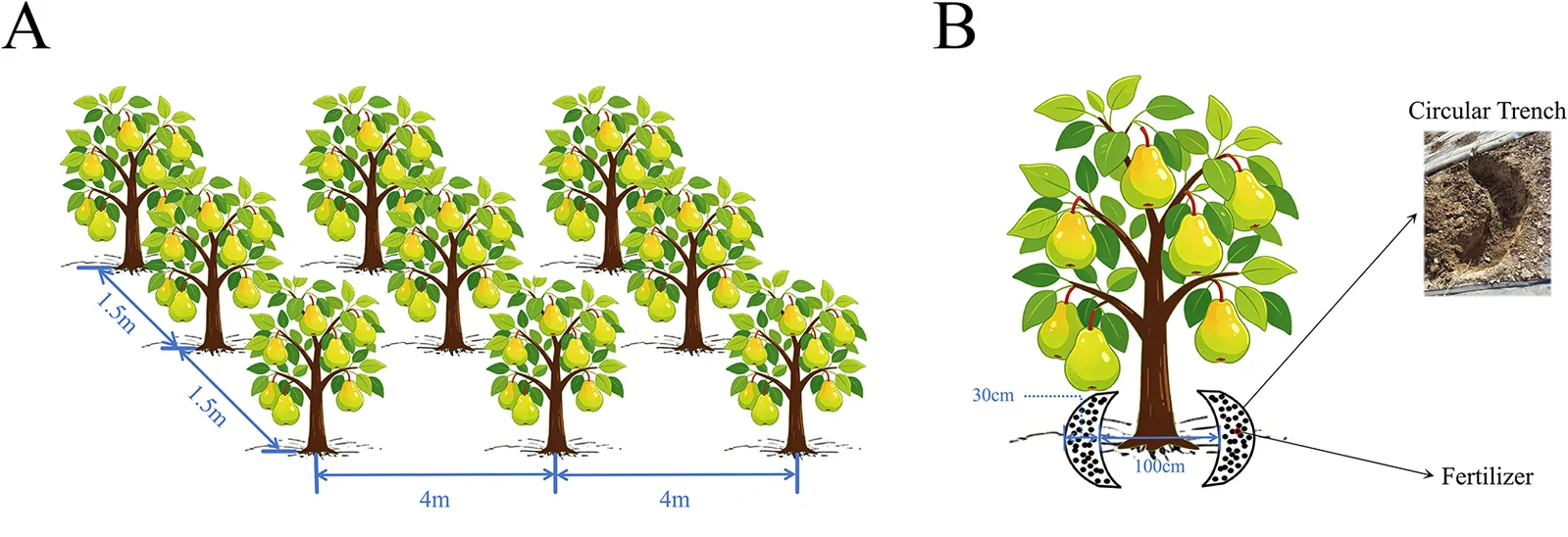
Schematic diagram of planting spacing for Korla fragrant pear **(A)**, and sketch of circular trench of Korla fragrant pear **(B)**.

**Table 1:** Experimental scheme for different fertilization treatments.

Irrigation stage	Irrigation time	Irrigation method	Irrigation frequency	Irrigation water amount/m^3^·hm^-2^	Nutrient application rate/kg·hm^-2^					
				W	N	P_2_O_5_	K_2_O			
							K0	K1	K2	K3
Winter irrigating	20 October	flood irrigation	1	1800	0	0	0	0	0	0
Spring irrigating	15 March	flood irrigation	1	1800	96	240	0	30	60	90
Fruit setting stage	5 May	drip irrigation	1	600	48	0	0	7.5	15	22.5
Early stage of fruit enlargement	25 May	drip irrigation	1	600	0	0	0	0	0	0
	15 June	drip irrigation	1	600	72	0	0	15	30	45
	5 July	drip irrigation	1	600	0	0	0	0	0	0
Late fruit expansion	25 July	drip irrigation	1	600	24	0	0	22.5	45	67.5
	15 August	drip irrigation	1	600	0	0	0	0	0	0
fruit ripening period	5 September	drip irrigation	1	600	0	0	0	0	0	0
Total			9	7800	240	240	0	75	150	225

### 2.3 Sample collection and processing

Soil samples: Soil samples were collected on August 3 during the fruit expansion stage. Three pear trees were randomly selected from each treatment. Soil was sampled from both sides of the fertilizer trench at depths of 0–20, 20–40, and 40–60 cm for each tree, and samples from the same soil layer were composited into one sample. The collected soil was gently broken up, thoroughly mixed, placed in resealable plastic bags, and transported to the laboratory in a dry-ice cooler. After removal of plant roots and large stones, the soil was passed through a 2 mm sieve and homogenized. Each sample was then divided into two portions. The portion used for soil enzyme assays was stored at low temperature until shipment to Nanjing Aoqing Biotechnology Co., Ltd. for determination of urease, catalase, sucrase, nitrate reductase, and alkaline phosphatase activities. The portion used for physicochemical analyses was air-dried indoors in the shade and passed through 1 mm and 0.25 mm sieves before analysis.

Root samples: Root samples were collected on August 3 during the fruit expansion stage using a soil auger with a diameter of 7 cm. Roots were sampled from three fruit trees per treatment along the inner side of the fertilizer trench. After removal of surface litter, root samples were collected within the canopy area from the east, south, west, and north sides of each tree at depths of 0–20, 20–40, and 40–60 cm. All sampled roots were fine roots. Root samples from the four directions within the same soil layer were rinsed with deionized water and composited. The roots were then divided into two equal portions and placed in cryovials containing standard FAA fixative, which were stored in a dry-ice cooler. One portion was sent to Nanjing Aoqing Biotechnology Co., Ltd. for root sectioning, while the other was returned to the laboratory for determination of root vitality.

### 2.4 Measurements and analytical methods

Determination of Soil Physicochemical Properties: Soil physicochemical properties were determined according to the methods described in *Soil and Agricultural Chemistry Analysis* (Bao, 2000). Soil pH was measured using a pH meter (FE28, Mettler Toledo, Shanghai, China) at a water-to-soil ratio of 5:1. Electrical conductivity (EC) was measured using a conductivity meter (DDS-11A, Youke Instruments, Shanghai, China). Soil organic matter was determined using the potassium dichromate–concentrated sulfuric acid external heating method. Total nitrogen was determined by sulfuric acid digestion followed by the Kjeldahl method. Alkali-hydrolyzable nitrogen was determined using the alkaline hydrolysis diffusion method. Available phosphorus was determined by extraction with 0.5 mol·L^-1^ NaHCO_3_ followed by the molybdenum–antimony colorimetric method, and available potassium was determined by extraction with 1 mol·L^-1^ ammonium acetate followed by flame photometry.

Soil Enzyme Activities: Urease, catalase, sucrase, nitrate reductase, and alkaline phosphatase activities were determined by Nanjing Aoqing Biotechnology Co., Ltd. using commercial assay kits (ST5200, ST5217, ST5221, ST5218, and ST5230, respectively). Urease activity was determined using the indophenol blue colorimetric method, with one unit of enzyme activity defined as the production of 1 μg ammonium nitrogen (NH_3_-N) per gram of soil per day. Catalase activity was determined by ultraviolet spectrophotometry, with one unit defined as the decomposition of 1 μmol H_2_O_2_ per gram of air-dried soil per day. Sucrase activity was determined colorimetrically, with one unit defined as the production of 1 mg reducing sugar per gram of soil per day. Nitrate reductase activity was determined colorimetrically, with one unit defined as the production of 1 μmol NO_2_^-^ per gram of soil per day. Alkaline phosphatase activity was determined colorimetrically, with one unit defined as the release of 1 μmol phenol per gram of soil per day at 37 °C.

Root Vitality: Root vitality was determined using the triphenyltetrazolium chloride (TTC) method (Duncan and Widholm, 2004). A 0.50 g root sample was placed in a 25 mL beaker. For the blank treatment, sulfuric acid was added before the root sample, while all other procedures were identical. A 10 mL mixture containing equal volumes of 0.4% TTC solution and phosphate buffer was added to completely immerse the roots, which were then incubated in the dark at 37 °C for 2 h. Subsequently, 2 mL of 1 mol·L^-1^ sulfuric acid was added to terminate the reaction, except in the blank treatment. The roots were removed, blotted dry with absorbent paper, and returned to the original beaker. Six milliliters of 95% ethanol was then added, and the samples were extracted for 15–20 min or sealed and left overnight until the red product was completely extracted. The extract was transferred to a 10 mL centrifuge tube, and the roots were washed two to three times with ethanol, with all wash solutions transferred to the same tube. The final volume was adjusted to 10 mL with 95% ethanol. The extract was centrifuged at 4,000 r·min^-1^ for 10 min and cooled, after which absorbance was measured at 485 nm using a spectrophotometer to calculate the amount of tetrazolium reduced.

Root Paraffin Sectioning and Structure Analysis: Root fixation, dehydration, paraffin embedding, sectioning, safranin–fast green staining, and digital slide preparation were performed by Nanjing Aoqing Biotechnology Co., Ltd. Root tissues were fixed in formalin–acetic acid–alcohol (FAA) solution, dehydrated through a graded ethanol series, cleared with xylene, and embedded in paraffin. Sections were cut to a thickness of 12–15 μm. After deparaffinization and rehydration, the sections were first stained with safranin for 10 min, rinsed with distilled water, and then stained with fast green for 2 min. The sections were subsequently rapidly differentiated with 1% hydrochloric acid–ethanol, dehydrated through a graded ethanol series, cleared with xylene, and mounted with neutral resin. After staining, lignified cell walls appeared red, whereas cellulose cell walls appeared green. For each treatment, three digital section images from each of the 0–20, 20–40, and 40–60 cm soil layers were obtained for quantitative analysis.

### 2.5 Data analysis

Experimental data were organized using Microsoft Excel 2016. One-way analysis of variance (one-way ANOVA) was performed using IBM SPSS Statistics 27 (IBM, USA), followed by Waller–Duncan multiple comparisons at a significance level of 0.05. Results are presented as the mean ± standard error (x ± SE). Pearson correlation analysis was used to assess the relationships among soil physicochemical properties, enzyme activities, and root vitality. Figures were generated using Origin 2024 (OriginLab, USA). ImageJ (NIH, USA) was used to count cells with cellulose and lignified cell walls in the corresponding root sections. NDP.view2 (Hamamatsu Photonics K.K., Japan) was used to measure cortex thickness, phloem thickness, xylem thickness, and section diameter, from which section area was calculated. Partial least squares structural equation modeling (PLS-SEM) was performed using SmartPLS 4 (SmartPLS GmbH, Germany), and 5,000 bootstrap resamples were used to evaluate the relationships among potassium fertilization gradients, soil physicochemical properties, soil enzyme activities, root traits, and yield.

## 3 Results

### 3.1 Effects of different potassium fertilization rates on soil physicochemical properties

Changes in the soil physicochemical properties of the Korla fragrant pear orchard under different potassium fertilization rates are shown in **Figure 3**. Overall, soil organic matter (SOM), total nitrogen (TN), alkali-hydrolyzable nitrogen (AN), available phosphorus (AP), available potassium (AK), pH, and electrical conductivity (EC) responded differently to potassium application across soil depths. AK increased progressively with increasing potassium application rate. The responses of AN and AP to potassium fertilization were mainly concentrated within the 0–40 cm soil layer. SOM generally increased under all potassium treatments, whereas TN did not differ significantly among treatments at any soil depth. Soil pH and EC generally decreased with increasing potassium application rate.

**Figure 3:**
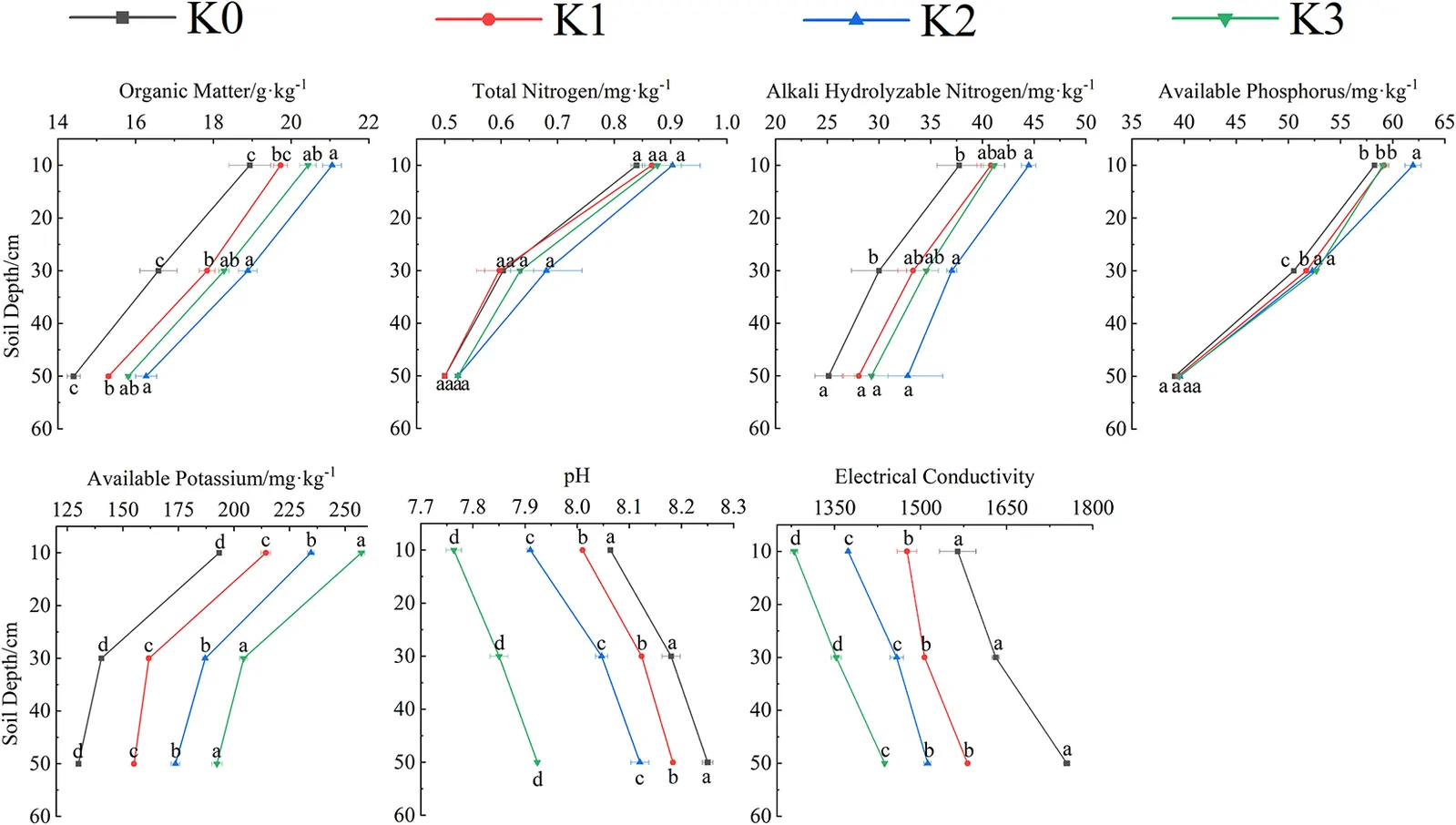
Effects of different gradient potassium fertilizer treatments on soil physical and chemical properties in Korla fragrant pear orchard. Data are presented as mean ± standard error (n = 3), with a significance level of *P* = 0.05. Different lowercase letters in the same column indicate significant differences (*P* < 0.05).

In the 0–20 cm soil layer, compared with K0, K1 significantly increased only AK. Under K2, AN, AP, AK, and SOM increased significantly by 17.84%, 6.33%, 21.38%, and 11.18%, respectively (*P* < 0.05). Under K3, AK and SOM increased significantly by 33.10% and 7.91%, respectively (*P* < 0.05), whereas TN, AN, and AP showed no significant changes. All potassium treatments significantly reduced soil pH and EC (*P* < 0.05), with the largest decreases observed under K3, at 3.72% and 18.21%, respectively.

In the 20–40 cm soil layer, potassium application had relatively pronounced effects on AP, AK, and SOM. Compared with K0, AK increased by 15.20%, 33.25%, and 45.61% under K1, K2, and K3, respectively, while SOM increased by 7.53%, 13.84%, and 10.16%, respectively (*P* < 0.05). AN increased significantly only under K2, by 23.44% (*P* < 0.05), whereas AP increased significantly under K1, K2, and K3 (*P* < 0.05), although the magnitude of increase was relatively small. All potassium treatments significantly reduced soil pH and EC (*P* < 0.05), with the greatest decreases again observed under K3, at 4.03% and 17.02%, respectively.

In the 40–60 cm soil layer, potassium application had more pronounced positive effects on AK and SOM, whereas TN, AN, and AP did not differ significantly from K0 under any potassium treatment. Compared with K0, AK increased significantly by 19.23%, 33.59%, and 47.95% under K1, K2, and K3, respectively (*P* < 0.05), showing a progressive increase with increasing potassium application rate. SOM increased significantly by 6.23%, 12.94%, and 9.76%, respectively (*P* < 0.05), with the largest increase observed under K2. All potassium treatments significantly reduced soil pH and EC (*P* < 0.05), and the largest decreases occurred under K3, by 3.94% and 18.09%, respectively.

### 3.2 Effects of different potassium fertilizer rates on soil enzyme activities

Changes in soil enzyme activities under different potassium fertilization rates are shown in **Figure 4**. Across the 0–60 cm soil profile, the activities of soil urease (URE), catalase (CAT), nitrate reductase (NR), sucrase (SUC), and alkaline phosphatase (ALP) gradually decreased with increasing soil depth. Compared with K0, URE, CAT, NR, SUC, and ALP generally showed increasing trends under potassium fertilization, although the magnitude of the response differed among enzymes.

**Figure 4:**
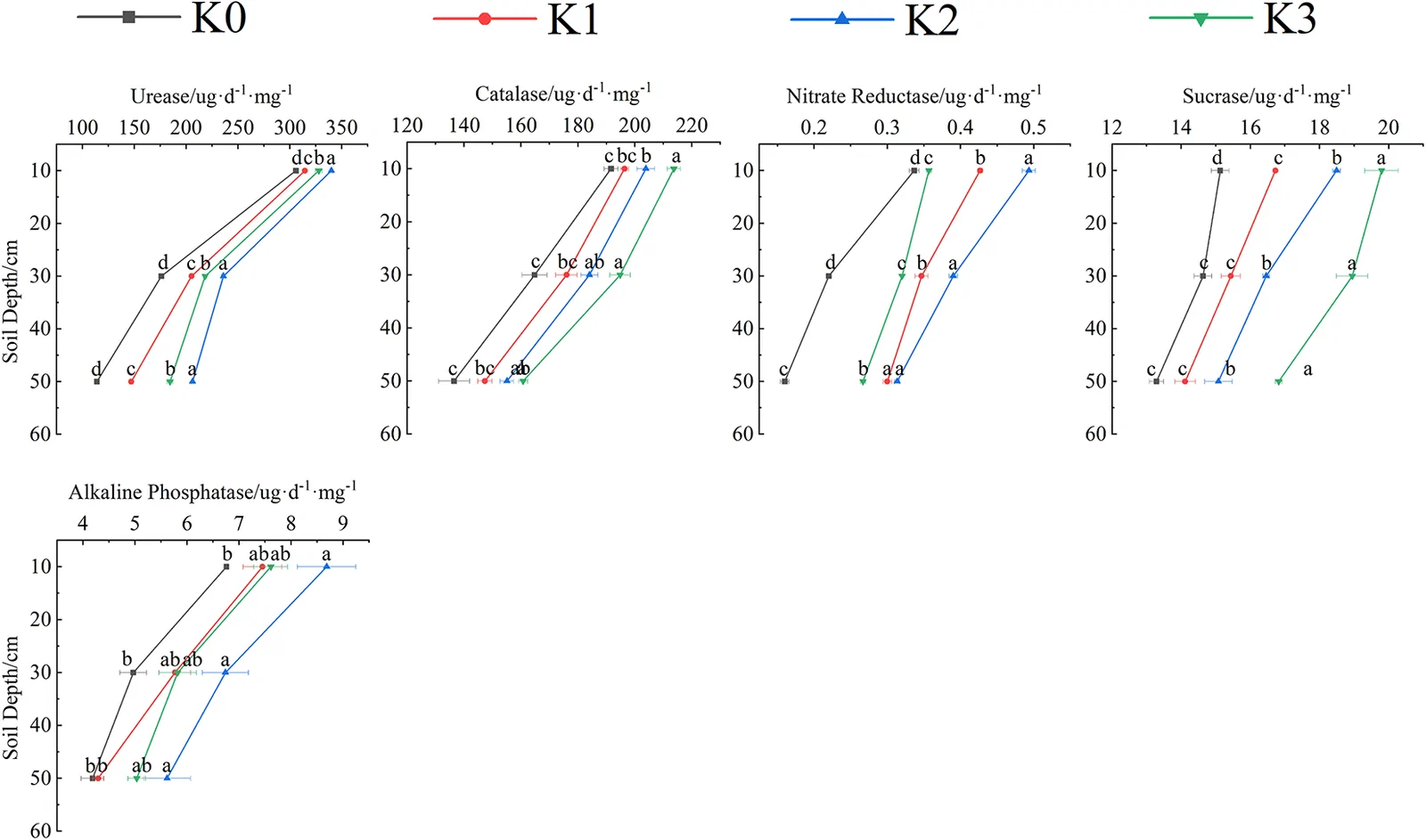
Effects of different gradient potassium fertilizer treatments on soil enzyme activities in Korla fragrant pear orchard. Data are presented as mean ± standard error (n = 3), with a significance level of *P* = 0.05. Different lowercase letters in the same column indicate significant differences (*P* < 0.05).

In the 0–20 cm soil layer, potassium application significantly increased URE, NR, and SUC activities. The largest increases in URE and NR were observed under K2, with values 11.20% and 46.12% higher than those under K0, respectively (*P* < 0.05). SUC showed the greatest increase under K3, reaching 30.88% above K0 (*P* < 0.05). CAT increased significantly only under K2 and K3, whereas ALP increased significantly only under K2, by 28.48% (*P* < 0.05).

In the 20–40 cm soil layer, the relative responses of most enzymes to potassium application were more pronounced than those in the 0–20 cm layer. URE and NR increased significantly under K1, K2, and K3, with the largest increases observed under K2, at 33.94% and 79.06% relative to K0, respectively (*P* < 0.05). CAT and SUC increased significantly mainly under K2 and K3, and SUC showed a relatively large increase under K3, by 29.49% (P < 0.05). ALP again increased significantly only under K2, by 35.59% (*P* < 0.05).

In the 40–60 cm soil layer, the relative responses of URE and NR to potassium application increased further. Under K2, URE and NR were significantly 80.82% and 95.10% higher than under K0, respectively (*P* < 0.05), and these increases were greater than those under K1 and K3. CAT and SUC continued to increase significantly mainly under K2 and K3, whereas ALP increased significantly only under K2, by 34.22% (*P* < 0.05). Overall, URE, NR, and ALP showed relatively pronounced responses under K2, whereas CAT and SUC increased more markedly at higher potassium application rates.

### 3.3 Effects of different potassium fertilization rates on root vitality of Korla fragrant pear

Changes in root vitality of Korla fragrant pear under different potassium fertilization rates are shown in **Figure 5**. Across the 0–60 cm soil profile, root vitality gradually decreased with increasing soil depth, and all potassium treatments showed significantly higher root vitality than K0 (*P* < 0.05). In the 0–20, 20–40, and 40–60 cm soil layers, K2 produced the greatest increases in root vitality, with values 54.19%, 50.60%, and 44.45% higher than those under K0, respectively. K3 showed the second-largest increases, whereas K1 produced relatively smaller increases. Overall, the enhancement of root vitality followed the order K2 > K3 > K1, and this response pattern was relatively consistent across the three soil depths.

**Figure 5:**
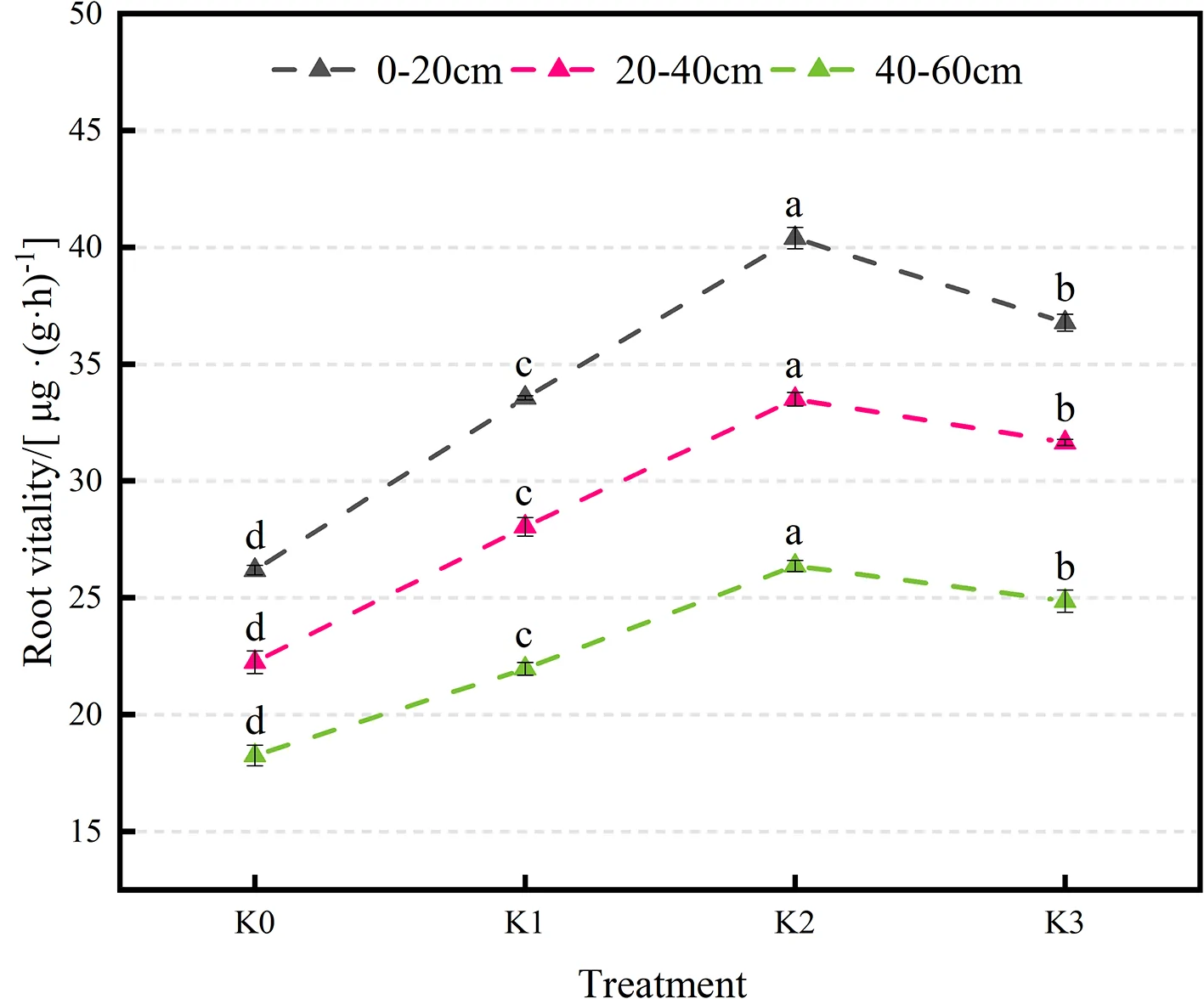
Effects of different gradient potassium fertilizer treatments on root vitality of Korla fragrant pear. Data are presented as mean ± standard error (n = 3), with a significance level of *P* = 0.05. Different lowercase letters in the same column indicate significant differences (*P* < 0.05).

### 3.4 Effects of different potassium fertilization rates on root anatomical structure of Korla fragrant pear

As shown in **Figure 6**, all root samples were fine roots, with cross-sectional areas of approximately 2.88 × 10^5^–1.57 × 10^6^ μm^2^, corresponding to equivalent diameters of approximately 0.61–1.41 mm. For root anatomical structure, we focused primarily on traits related to cortex thickness, the proportions of different cell types in the xylem and phloem, vessel area, and root cross-sectional area.

**Figure 6:**
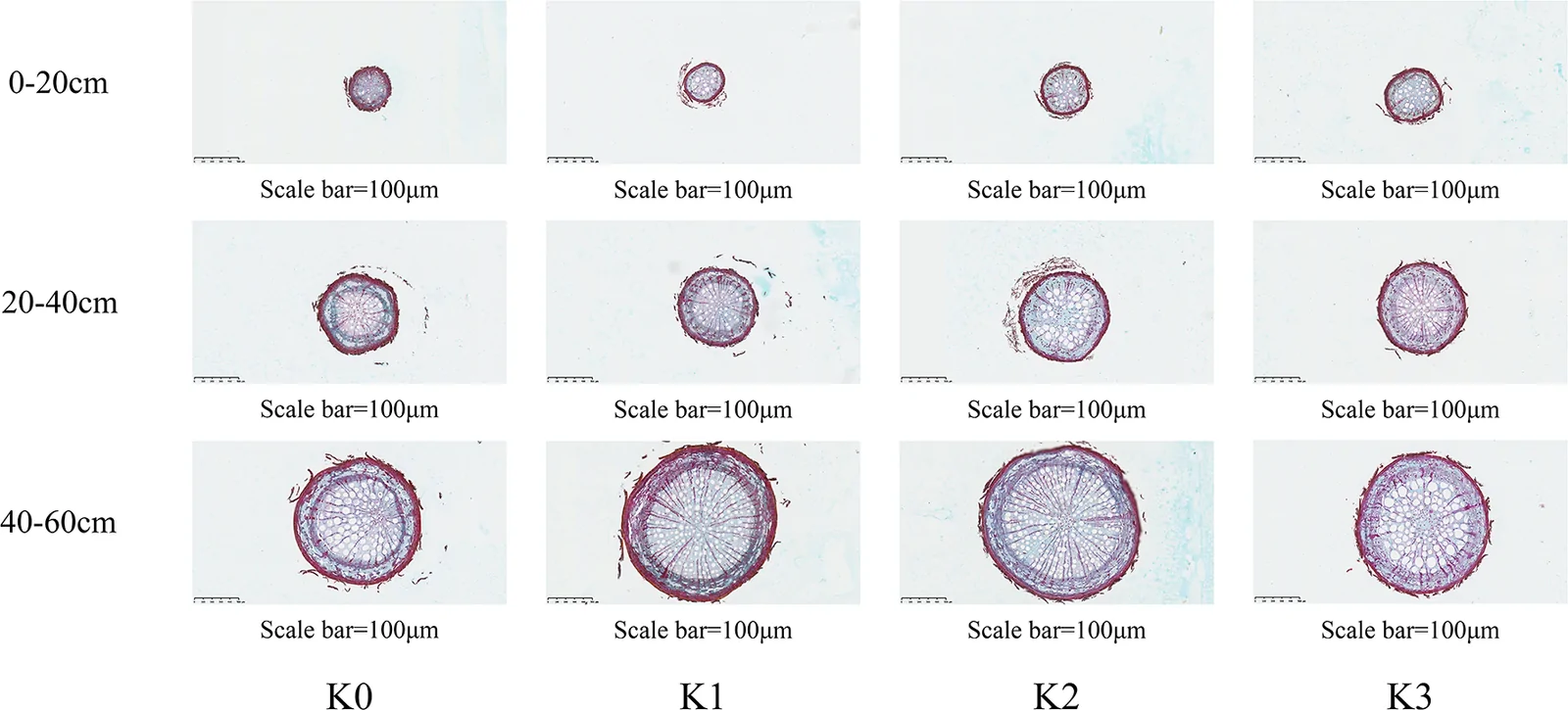
Paraffin sections of Korla fragrant pear roots under different gradient potassium fertilizer treatments.

#### 3.4.1 Effects of different potassium fertilization rates on root cortical thickness

As shown in **Figure 7**, root cortex thickness changed only slightly with increasing soil depth across the 0–60 cm soil profile, whereas it was generally lower under potassium fertilization than under K0. In the 0–20 cm soil layer, K1, K2, and K3 all significantly reduced root cortex thickness, with the greatest decrease observed under K2, at 35.15%. In the 20–40 cm soil layer, all potassium treatments likewise significantly reduced cortex thickness, with K2 producing the largest decrease of 42.71%. In the 40–60 cm soil layer, only K2 and K3 differed significantly from K0, decreasing cortex thickness by 21.56% and 25.95%, respectively, whereas K1 showed no significant difference (*P* < 0.05). Overall, potassium application generally reduced root cortex thickness across the 0–60 cm soil profile, with K2 showing relatively pronounced decreases at all soil depths.

**Figure 7:**
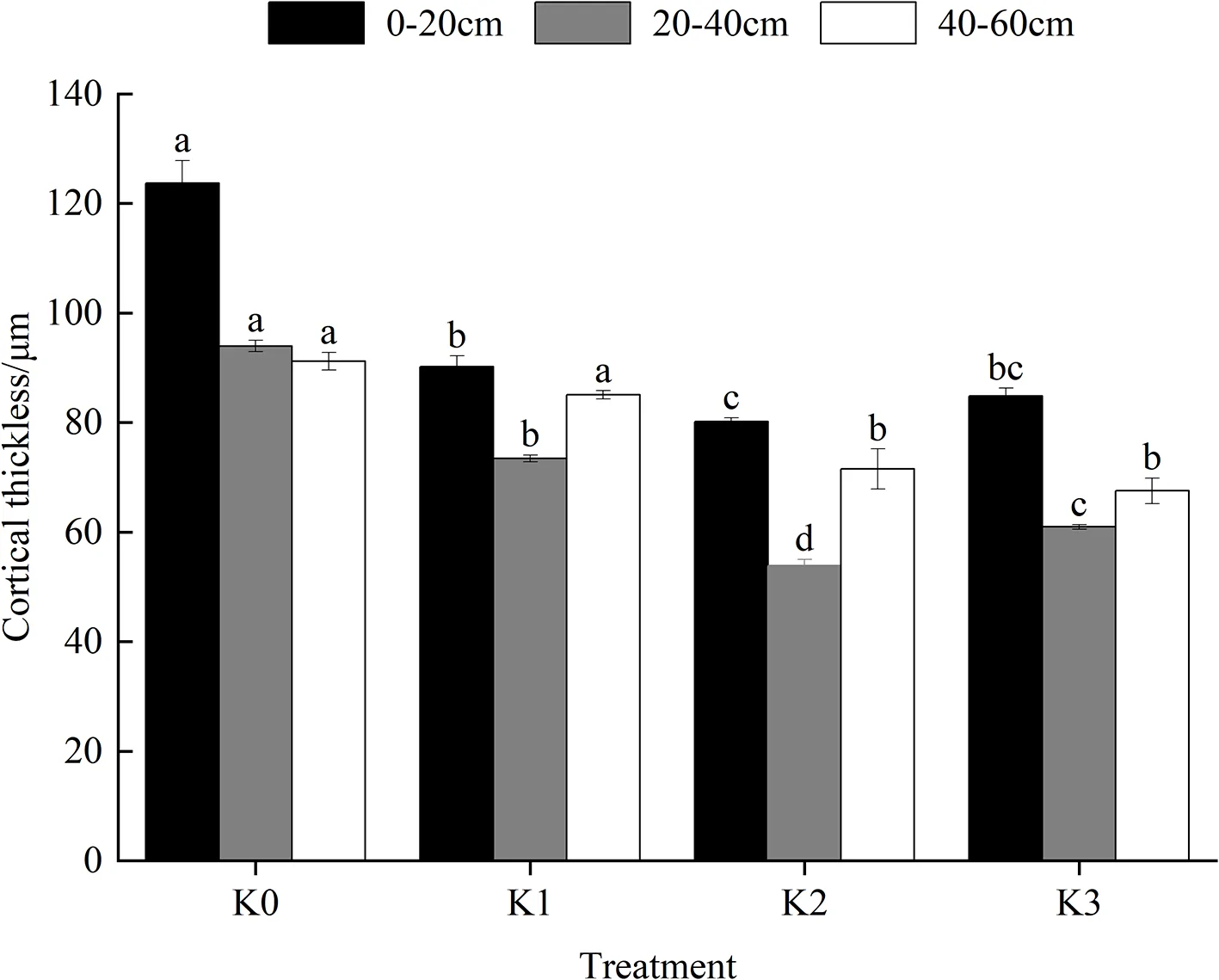
Effects of different gradient potassium fertilizer treatments on root cortex thickness of Korla fragrant pear. Data are presented as mean ± standard error (n = 3), with a significance level of *P* = 0.05. Different lowercase letters in the same column indicate significant differences (*P* < 0.05).

#### 3.4.2 Effects of different potassium fertilization rates on the proportion of different cell types in xylem and phloem of roots

As shown in **Figure 8**, across the 0–60 cm soil profile, all potassium fertilization treatments significantly increased the proportion of cells with cellulose cell walls in the root phloem compared with K0 (*P* < 0.05). Under K2, the proportions in the 0–20, 20–40, and 40–60 cm soil layers were 91.64%, 94.83%, and 90.57%, respectively, representing increases of 34.06, 32.63, and 37.22 percentage points, respectively, compared with K0. Overall, the increases were greater than those under K1 and K3.

**Figure 8:**
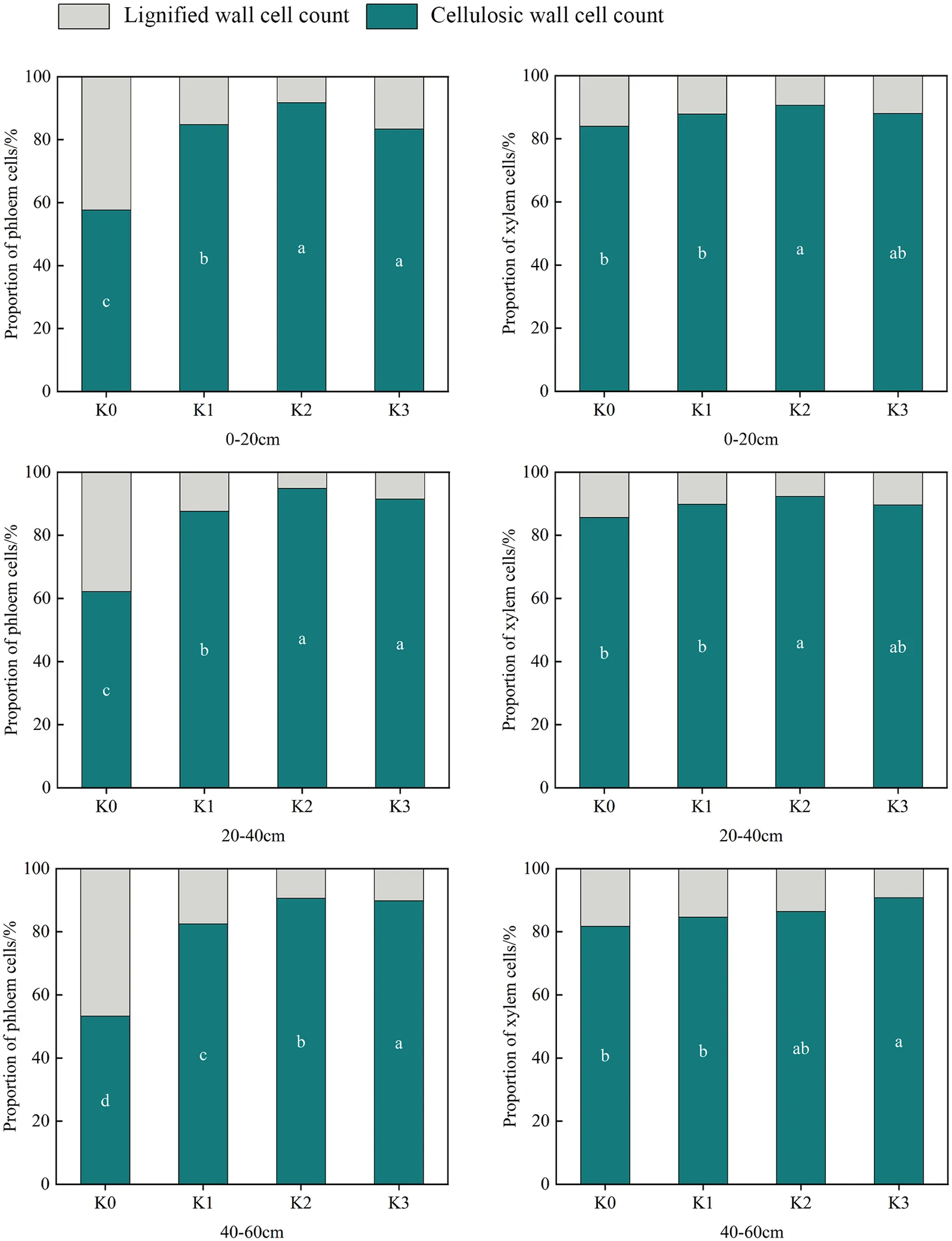
Effects of different gradient potassium fertilizer treatments on root xylem and phloem cells of Korla fragrant pear. Data are presented as mean ± standard error (n = 3), with a significance level of *P* = 0.05. Different lowercase letters in the same column indicate significant differences (*P* < 0.05).

In contrast, the proportion of cells with cellulose cell walls in the root xylem showed a relatively weaker response to potassium application. In the 0–20 and 20–40 cm soil layers, only K2 was significantly higher than K0, with proportions of 89.92% and 92.38%, respectively, representing increases of 4.41 and 6.87 percentage points, respectively, compared with K0. In the 40–60 cm soil layer, only K3 showed a significant difference, with a proportion of 90.84%, representing an increase of 9.04 percentage points compared with K0 (*P* < 0.05). Although the other treatments showed varying degrees of increase, none differed significantly from K0.

#### 3.4.3 Effects of different potassium fertilization rates on vessel area and cross-sectional area of roots

Changes in root vessel area and total cross-sectional area of Korla fragrant pear under different potassium fertilization rates are shown in **Figure 9**. In the 0–20 cm soil layer, both vessel area and total cross-sectional area were significantly higher under K1, K2, and K3 than under K0 (*P* < 0.05). Among these treatments, vessel area showed a relatively large increase under K2, reaching 42.34%, whereas total cross-sectional area showed the greatest increase under K3, reaching 75.33%.In the 20–40 cm soil layer, vessel area increased significantly only under K2 and K3, by 197.87% and 299.68%, respectively, compared with K0, whereas no significant difference was observed under K1. Total cross-sectional area increased significantly under all three potassium treatments, with the greatest increase observed under K3, at 72.47% (*P* < 0.05).In the 40–60 cm soil layer, both vessel area and total cross-sectional area were significantly higher under all potassium treatments than under K0 (*P* < 0.05). The greatest increases in both traits were observed under K3, with vessel area and total cross-sectional area increasing by 483.50% and 64.54%, respectively. Overall, potassium application generally increased root vessel area and total cross-sectional area, although the magnitude of these responses varied markedly among soil depths and potassium application rates.

**Figure 9:**
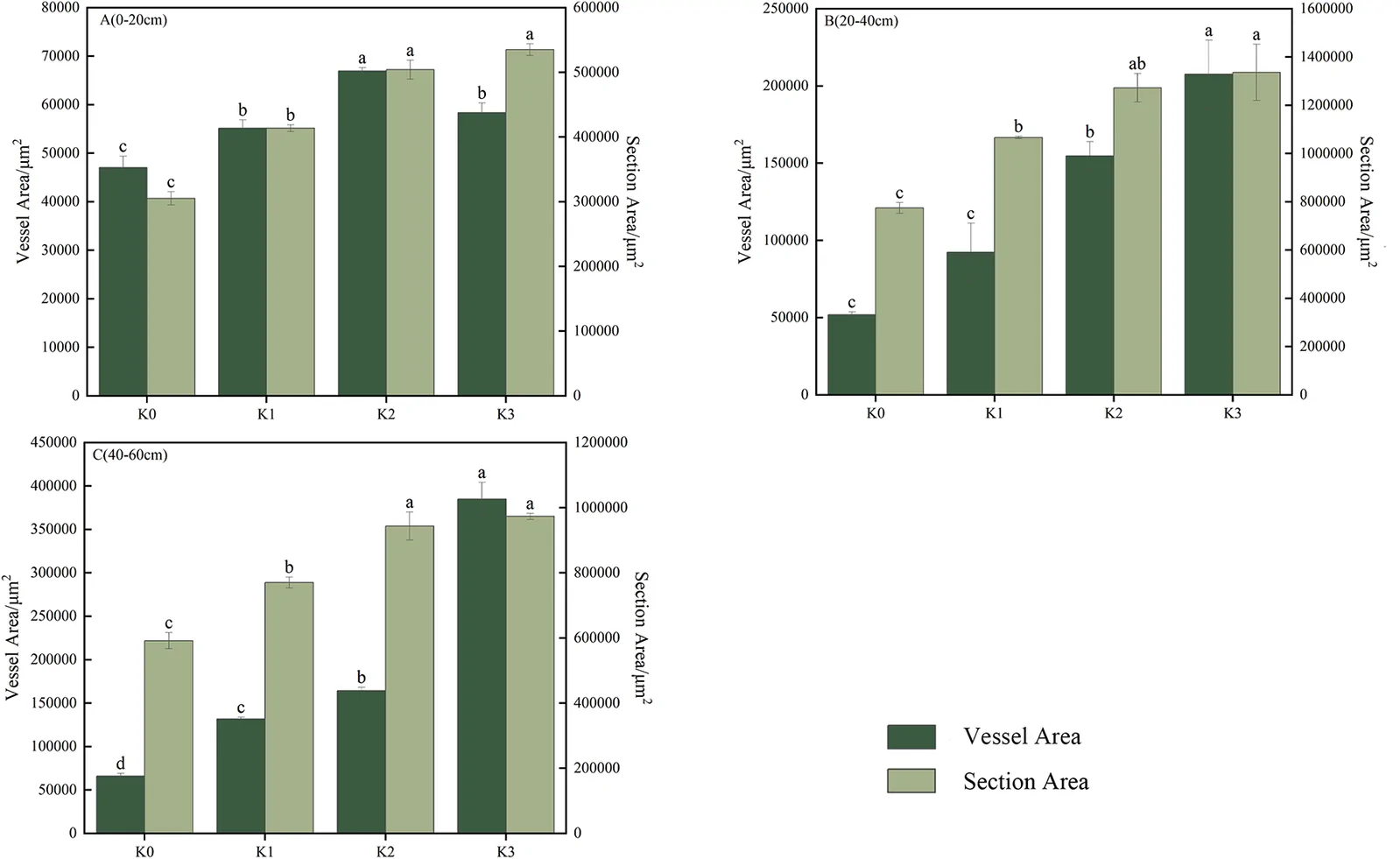
Effects of different gradient potassium fertilizer treatments on root vessel and slice area of Korla fragrant pear. Data are presented as mean ± standard error (n = 3), with a significance level of *P* = 0.05. Different lowercase letters in the same column indicate significant differences (*P* < 0.05).

### 3.5 Correlations among soil physicochemical properties, enzyme activities, and root structural traits under different potassium fertilization rates

Mantel tests were conducted to examine the relationships among soil physicochemical properties, enzyme activities, and root structural traits under different potassium fertilization rates (**Figure 10**). The results showed that these variables were correlated to varying degrees across different soil depths, and the strength of these correlations changed markedly with soil depth.

**Figure 10:**
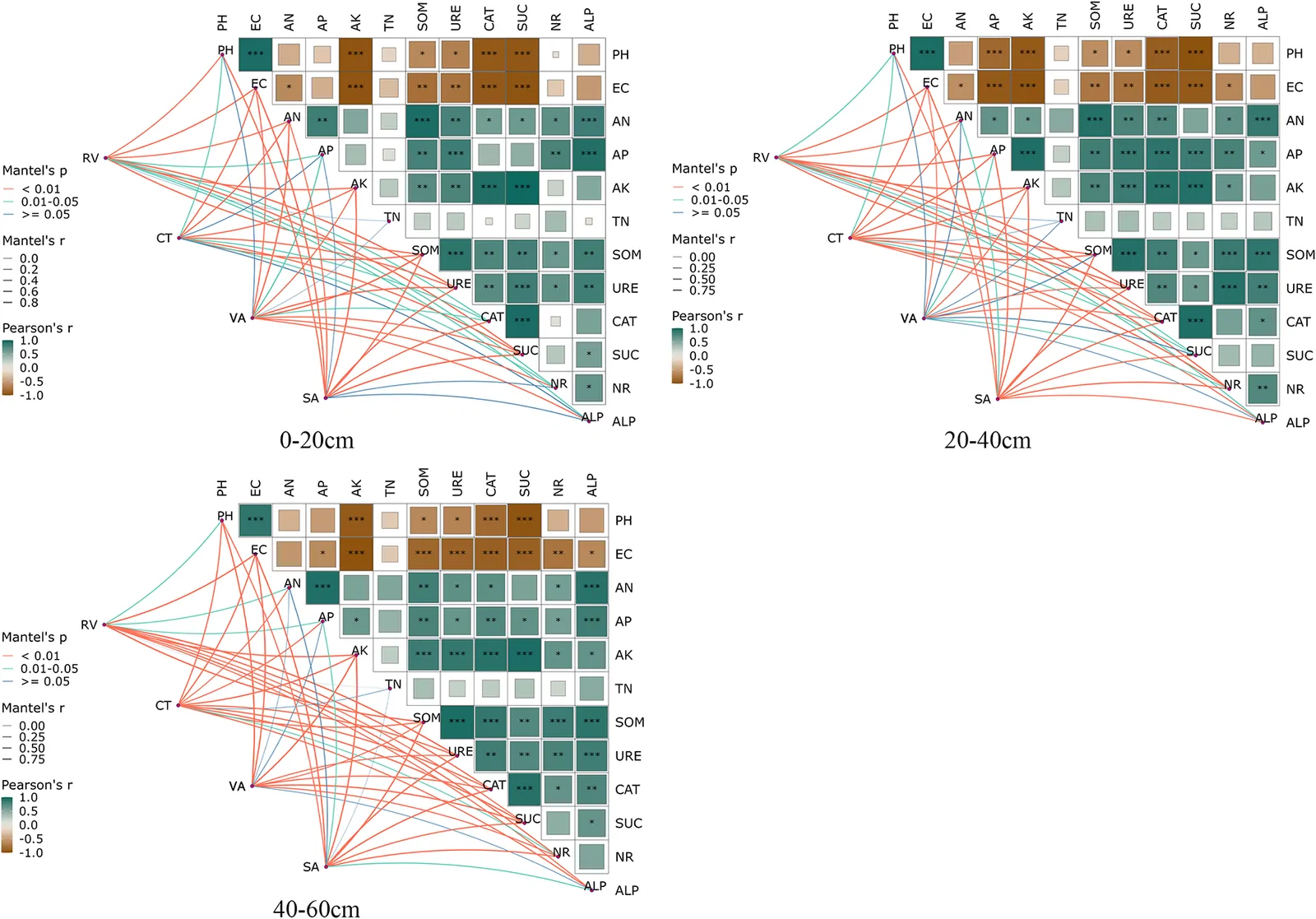
Mantel test of soil physical and chemical properties, enzyme activity and root vitality under different gradient potassium fertilizer treatments. Darker brown indicates a stronger negative correlation, while green signifies a stronger positive correlation. * represents significant correlation (*P* < 0.05), **, *** represents extremely significant correlation (*P* < 0.01). The thickness of lines connecting yield, single fruit weight, environmental factors and enzyme activities indicates the correlation degree. The thicker the line, the stronger the correlation, and vice versa. The color of lines between nodes and environmental factors represents the *P*-value. Soil pH, soil pH; EC, electrical conductivity; AN, alkali-hydrolyzed nitrogen content; AP, available phosphorus content; AK, available potassium content; TN, total nitrogen content; SOM, organic matter content; URE, urease activity; CAT, catalase activity; SUC, sucrase activity; NR, nitrate reductase activity; ALP, alkaline phosphatase activity; RV, root vitality; CT, cortical thickness; VA, vessel area; SA, section area.

In the 0–20 cm soil layer, root vitality (RV) showed the strongest correlation with URE (Mantel *r* = 0.789, *P* = 0.001) and was also strongly correlated with SOM, AK, EC, and SUC, with Mantel r values of 0.673, 0.661, 0.620, and 0.690, respectively (*P* < 0.003). Cortex thickness (CT) was mainly correlated with SOM, SUC, URE, and AK, with the strongest correlation observed with SOM (*r* = 0.675, *P* = 0.001). Vessel area (VA) was strongly correlated with SOM, AN, and URE, with Mantel *r* values of 0.851, 0.799, and 0.778, respectively (*P* = 0.001). Total cross-sectional area (SA) was strongly correlated with SUC, AK, and EC, with r values of 0.873, 0.842, and 0.835, respectively (*P* = 0.001).

In the 20–40 cm soil layer, the correlation between RV and URE became stronger (*r* = 0.947, *P* = 0.001). RV was also strongly correlated with AP, NR, SOM, and AK, with r values of 0.853, 0.834, 0.808, and 0.786, respectively. CT showed the strongest correlation with URE (*r* = 0.977, *P* = 0.001) and remained highly correlated with AK, NR, AP, and SOM. The correlation coefficients of VA with CAT, AK, and AP were 0.472, 0.468, and 0.416, respectively. SA was strongly correlated with AP, EC, AK, and URE, with Mantel r values of 0.829, 0.809, 0.794, and 0.789, respectively (*P* < 0.001).

In the 40–60 cm soil layer, RV remained strongly correlated with URE, SOM, EC, and AK. Among these variables, URE showed the highest Mantel r value (*r* = 0.940, *P* = 0.001), while the r values for SOM, EC, and AK were 0.894, 0.854, and 0.790, respectively. CT was strongly correlated with SUC and AK, with r values of 0.819 and 0.817, respectively (*P* = 0.001). The correlations of VA with pH, AK, and EC became markedly stronger, with Mantel r values of 0.915, 0.914, and 0.867, respectively (*P* = 0.001), and VA was also strongly correlated with SUC (*r* = 0.848, *P* = 0.001). SA was mainly strongly correlated with EC, AK, SOM, CAT, and URE, with r values of 0.917, 0.876, 0.850, 0.849, and 0.844, respectively (*P* = 0.001).

Across soil depths, URE, AK, EC, and SOM showed relatively consistent correlations with multiple root traits, although the strength of these correlations varied markedly along the soil profile. In particular, the Mantel r between RV and URE increased from 0.789 in the 0–20 cm layer to 0.947 and 0.940 in the 20–40 and 40–60 cm layers, respectively. The relationships between VA and soil physicochemical properties and enzyme activities varied more clearly with soil depth. In the 0–20 cm layer, VA was mainly strongly correlated with SOM, AN, and URE, whereas in the 40–60 cm layer, it showed stronger correlations with pH, AK, and EC. These results indicate that the associations among soil physicochemical properties, enzyme activities, and root traits differed markedly among soil depths.

### 3.6 Effects of different potassium fertilization rates on yield

As shown in **Figure 11**, compared with K0, K1, K2, and K3 all significantly increased individual fruit weight (*P* < 0.05), with the greatest increase observed under K2, at 18.19%. The number of fruits per tree under K2 was significantly 24.81% higher than that under K0 (*P* < 0.05), whereas no significant differences were observed between K1 or K3 and K0. Yield generally increased and then decreased with increasing potassium application rate. Compared with K0, yield increased significantly by 47.36% under K2 and by 28.93% under K3 (*P* < 0.05). Overall, under the conditions of this study, K2 showed relatively favorable performance in individual fruit weight, fruit number per tree, and yield.

**Figure 11:**
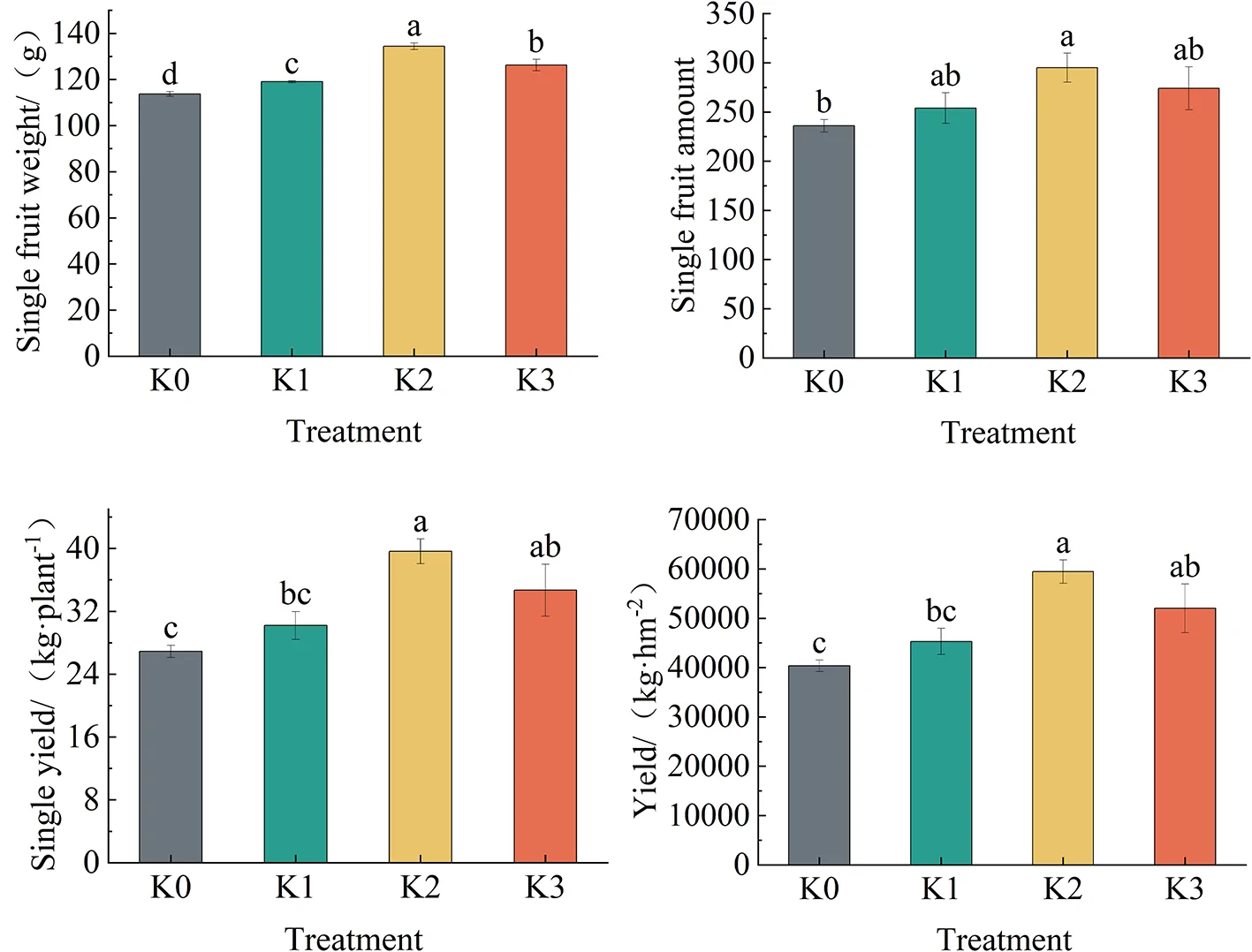
Effects of different potassium fertilizer rates on Korla fragrant pear orchard yield. Data are presented as mean ± standard error (n = 3), with a significance level of *P* = 0.05. Different lowercase letters in the same column indicate significant differences (*P* < 0.05).

### 3.7 Principal component analysis of soil physicochemical properties, enzyme activities, root traits, and yield under different potassium fertilization rates

Principal component analysis (PCA) was performed on soil physicochemical properties, enzyme activities, root structural traits, and Korla fragrant pear yield under different potassium fertilization rates, and the results are shown in **Figure 12**. The first two principal components explained most of the total variation at each soil depth, while the direction and magnitude of variable loadings in the principal component space varied with soil depth.

**Figure 12:**
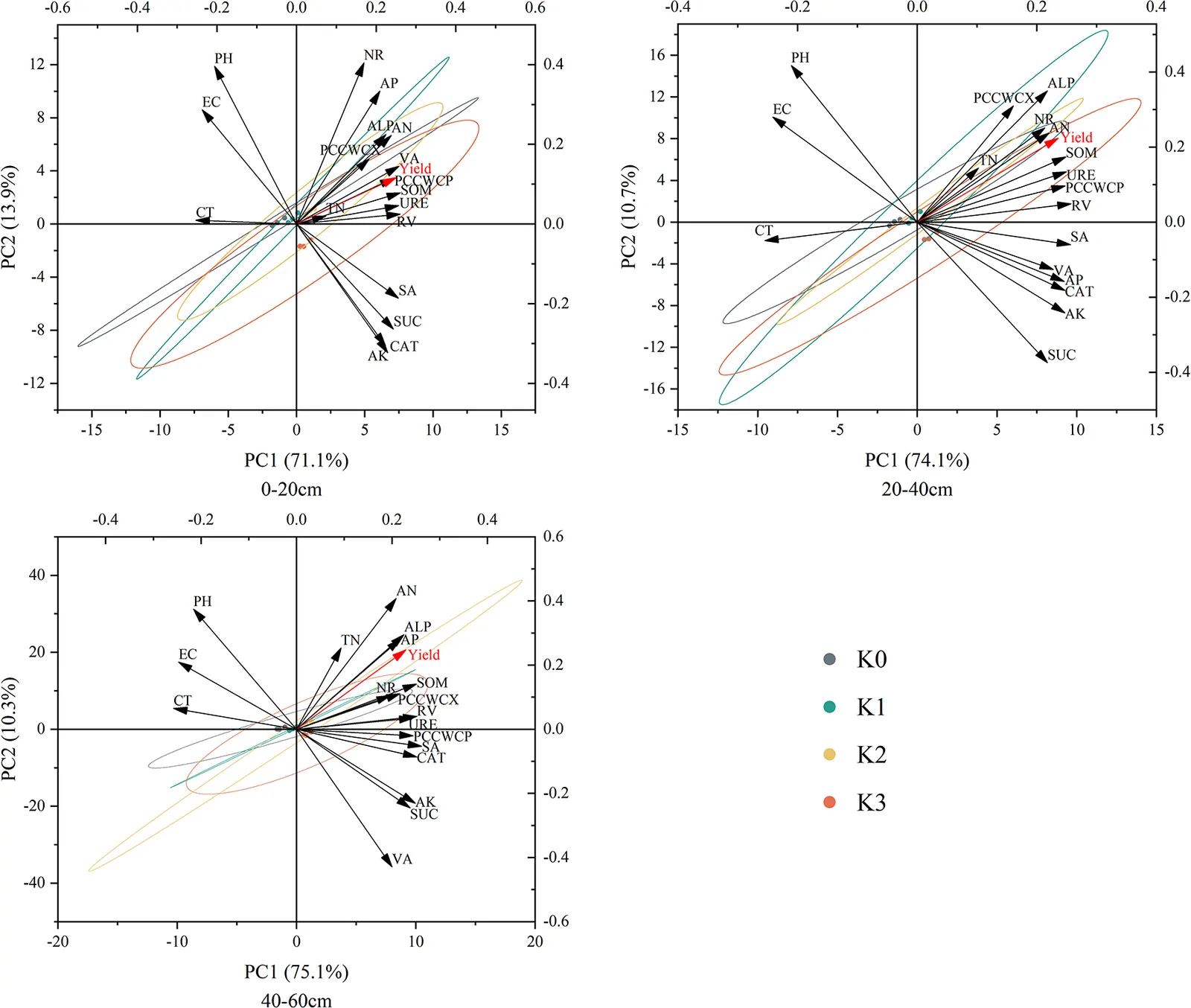
Principal component analysis of soil physical and chemical properties, enzyme activity, root characteristics and yield under different gradient potassium fertilizer treatments. Soil pH, soil pH; EC, electrical conductivity; AN, alkali-hydrolyzed nitrogen content; AP, available phosphorus content; AK, available potassium content; TN, total nitrogen content; SOM, organic matter content; URE, urease activity; CAT, catalase activity; SUC, sucrase activity; NR, nitrate reductase activity; ALP, alkaline phosphatase activity; RV, root vitality; CT, cortical thickness; VA, vessel area; SA, section area; PCCWCX, The proportion of cellulose cell wall cells in xylem; PCCWCP, The proportion of cellulose cell wall cells in phloem.

In the 0–20 cm soil layer, PC1 and PC2 together explained 85.00% of the total variation, accounting for 71.10% and 13.90%, respectively. On PC1, SOM, AN, AK, AP, URE, SUC, ALP, CAT, RV, VA, SA, and the proportion of cellulose cell wall cells in the phloem (PCCWCP) showed relatively strong positive loadings (loading> 0.20), whereas pH, EC, and CT showed relatively strong negative loadings (loading> 0.20). On PC2, NR, AN, ALP, and AP showed relatively strong positive loadings (loading> 0.21). The loadings of Yield on PC1 and PC2 were 0.25 and 0.12, respectively, indicating that yield was more closely aligned with the overall variation represented by PC1 in the principal component space. The treatments showed a certain degree of separation in the PCA space, with K2 mainly distributed in the higher positive region of PC1, followed by K3, while K1 and K0 were located relatively closer to the lower PC1 region.

In the 20–40 cm soil layer, PC1 and PC2 together explained 84.80% of the total variation, contributing 74.10% and 10.70%, respectively. On PC1, SOM, AN, AP, AK, URE, CAT, SUC, NR, ALP, RV, SA, PCCWCP, and VA all showed relatively strong positive loadings, whereas pH, EC, and CT showed relatively strong negative loadings (loading> 0.20). On PC2, ALP, NR, and the proportion of cellulose cell wall cells in the xylem (PCCWCX) showed relatively strong positive loadings (loading> 0.24). The loadings of Yield on PC1 and PC2 were 0.24 and 0.22, respectively, indicating that yield was associated with the integrated variation represented by both principal components. The treatment distribution in the PCA space generally showed K2 in the higher PC1 region, followed by K3, while K1 and K0 were located relatively lower along PC1.

In the 40–60 cm soil layer, PC1 and PC2 together explained 85.40% of the total variation, accounting for 75.10% and 10.30%, respectively. On PC1, SOM, AN, AP, AK, URE, CAT, SUC, ALP, RV, SA, PCCWCP, and PCCWCX showed relatively strong positive loadings (loading> 0.20), whereas pH, EC, and CT showed relatively strong negative loadings (loading> 0.21). On PC2, AN, AP, TN, and ALP showed relatively strong positive loadings (loading> 0.24), whereas VA showed a strong negative loading (loading> 0.42). The loadings of Yield on PC1 and PC2 were 0.23 and 0.25, respectively, indicating that yield in the deeper soil layer was associated with the integrated variation represented by both principal components. The distribution of treatments differed from that in the upper soil layers, with K3 showing a more pronounced position in the positive PC1 region, followed by K2.

Overall, PC1 at all three soil depths was mainly characterized by positive loadings of soil nutrients, enzyme activities, and most root-related traits, whereas pH, EC, and CT were primarily associated with negative loadings. However, the major variables associated with PC2 and the distribution of treatments varied with soil depth, indicating clear vertical differences in the multivariate associations among soil physicochemical properties, enzyme activities, root traits, and yield.

### 3.8 PLS-SEM analysis of the effects of potassium fertilization

To further integrate the relationships among soil physicochemical properties, soil enzyme activities, root traits, and Korla fragrant pear yield under different potassium application rates, a partial least squares structural equation model (PLS-SEM) was constructed based on the Mantel test and PCA results (**Figure 13**). Model evaluation showed that the average variance extracted (AVE) values for Root traits, Soil enzymes, Soil properties, and Yield were 0.510, 0.891, 0.813, and 0.716, respectively, while the corresponding composite reliability (CR) values were 0.798, 0.970, 0.751, and 0.882, indicating generally acceptable internal consistency and convergent validity. The inner variance inflation factor (VIF) values ranged from 1.000 to 1.140, indicating no apparent structural collinearity. Within the Root traits construct, the outer loadings of RV, PCCWCP, and PCCWCX were 0.756, 0.898, and 0.673, respectively. The outer loading of VA was relatively low (0.455), but remained significant based on bootstrap testing (*P* = 0.006) and was therefore retained because of its biological relevance as an important indicator of root vascular structure. The heterotrait-monotrait ratio (HTMT) results indicated a certain degree of discriminant validity among most constructs, although the HTMT value between Soil properties and Soil enzymes was 0.993, suggesting substantial empirical overlap between the two constructs. The standardized root mean square residual (SRMR) values were 0.222 for the saturated model and 0.238 for the estimated model, indicating limitations in overall model fit. Therefore, subsequent interpretation focused primarily on path coefficients and R^2^ values to characterize the structural associations among the variable groups. Detailed statistics are provided in the **Supplementary Material**.

**Figure 13:**
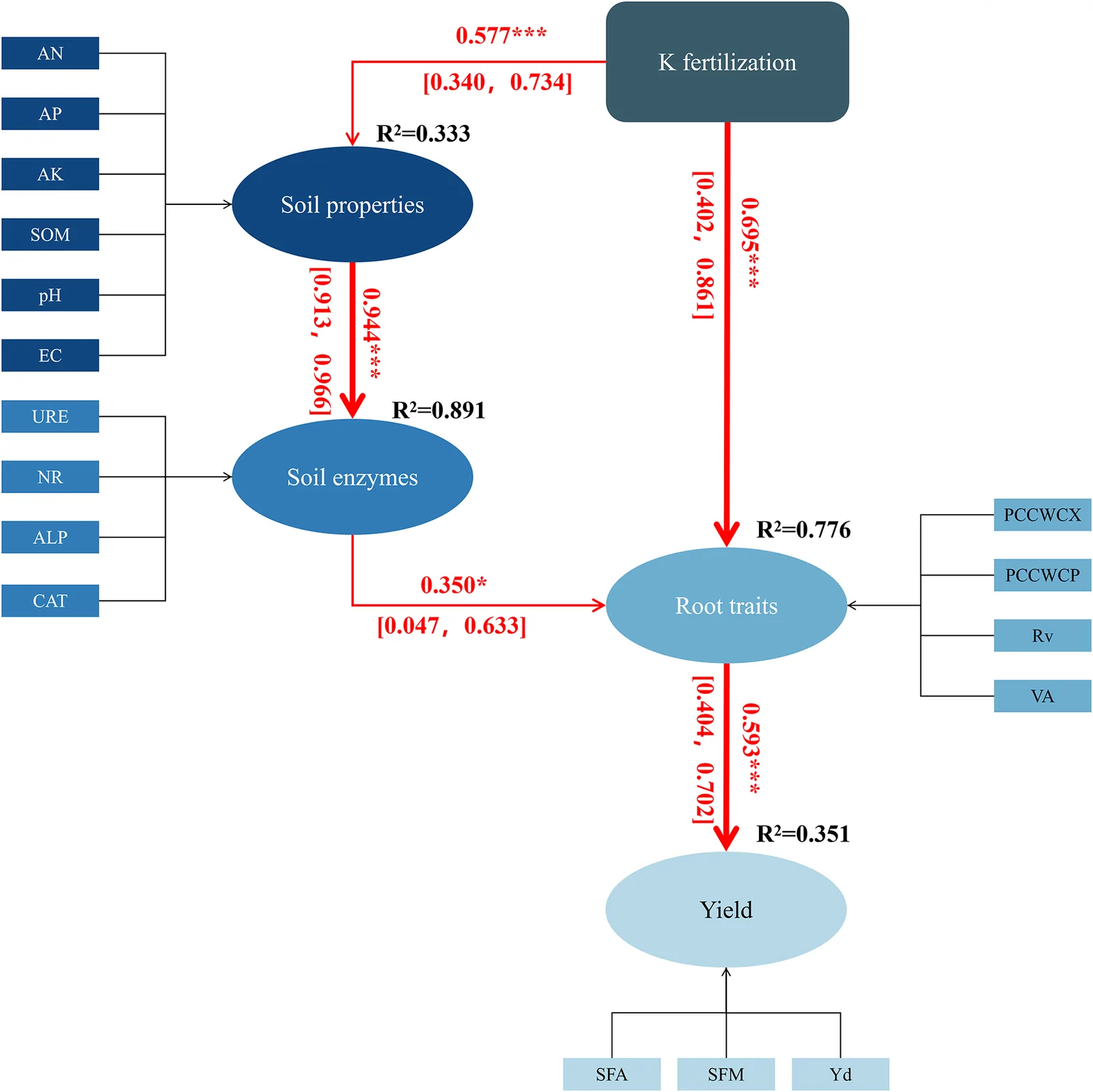
Partial least squares structural equation model of root-soil interactions and yield relationships under different potassium fertilization rates. Red arrows indicate positive structural paths in the model; Values beside the arrows represent standardized path coefficients, with 95% bias-corrected bootstrap confidence intervals shown in brackets based on 5,000 bootstrap samples; *, ** and *** indicate significant differences at the *P* < 0.05, *P* < 0.01 and *P* < 0.001 levels, respectively; R2 represents the proportion of variance explained for each endogenous construct.

The R^2^ values for Soil properties, Soil enzymes, Root traits, and Yield were 0.333, 0.891, 0.776, and 0.351, respectively. The model therefore explained a relatively high proportion of the variance in soil enzyme activities and root traits, whereas its explanatory power for yield was comparatively limited. Path analysis showed that the potassium fertilization gradient was positively associated with both Soil properties (β = 0.577, *P* < 0.001) and Root traits (β = 0.695, *P* < 0.001). Soil properties were also positively associated with Soil enzymes (β = 0.944, *P* < 0.001). Soil enzymes were positively associated with Root traits (β = 0.350, *P* < 0.05), and Root traits were positively associated with Yield (β = 0.593, *P* < 0.001). Overall, the PLS-SEM results were broadly consistent with the multivariate association patterns identified by the Mantel tests and PCA, revealing a continuous structural association of “potassium fertilization gradient–soil physicochemical properties–soil enzyme activities–root traits–yield.”

## 4 Discussion

### 4.1 Effects of different potassium fertilization rates on soil physicochemical properties

Soil physicochemical properties play an important role in the growth and development of fruit trees, and appropriate potassium fertilization can promote soil nutrient accumulation and improve plant nutrient use efficiency (Wen et al., 2021). In this study, compared with the no-potassium treatment, all potassium treatments increased soil organic matter content within the 0–60 cm soil profile of the Korla fragrant pear orchard. Overall, the K2 treatment performed relatively well, whereas further increases in potassium application resulted in a decline in soil organic matter content. This pattern may be related to the combined effects of potassium fertilization on plant growth, microbial activity, decomposition of organic residues, and soil nutrient cycling (Kaewu, 2024). Potassium application had no significant effect on soil total nitrogen content, although numerical increases were observed. This may be because potassium fertilization can modify root morphology, stimulate the release of root exudates, and enhance microbial activity, thereby affecting organic carbon inputs and microbial nitrogen transformation (Xu et al., 2021). Walia et al. (2024) reported that soil total nitrogen remained relatively stable even under markedly different potassium application rates, suggesting that soil total nitrogen is more strongly regulated by nitrogen inputs and long-term nitrogen cycling processes. In the present study, available potassium increased continuously with increasing potassium application rate, indicating that external potassium fertilizer effectively replenished soil available K and enhanced soil K-supplying capacity (Zhao et al., 2014). In contrast, the responses of alkali-hydrolyzable nitrogen and available phosphorus did not continue to increase with increasing potassium application rate, and their positive responses generally weakened under the higher K3 treatment. This asynchronous response may be related to the alkaline soil chemical conditions of the study area. Under such conditions, calcium- and carbonate-related processes in the soil may affect phosphorus availability through adsorption, precipitation, and other processes (Geng et al., 2022). Meyer et al. (2020) showed that large inputs of external K and changes in the relative proportions of K^+^ and Ca^2+^ at exchange sites can affect the adsorption behavior and availability of phosphate anions (H_2_PO_4_^-^ or HPO_4_^2-^). Therefore, under higher potassium application rates, an increase in soil K^+^ concentration may alter the ionic composition of the rhizosphere and the cation-exchange environment, thereby further affecting the adsorption–desorption balance of P. This may partly explain why AK continued to increase under K3 while AP did not increase proportionally. However, the specific mechanisms remain unclear and require further investigation. In addition, most soil nutrients in this study gradually decreased with increasing soil depth, showing a clear pattern of surface-layer enrichment. This vertical distribution may be associated with litter decomposition near the soil surface, inputs of root exudates, and the movement of water and nutrients under drip irrigation (Mao et al., 2024). Liu et al. (2025) found marked differences in soil nutrient status among different Korla fragrant pear production areas, indicating that regional soil background conditions and long-term orchard management may jointly influence orchard soil fertility characteristics.

### 4.2 Effects of different potassium fertilization rates on soil enzyme activities

In this study, potassium application generally increased the activities of soil urease, nitrate reductase, alkaline phosphatase, sucrase, and catalase, although the responses of individual enzymes varied among potassium application rates. Potassium fertilization effectively increased soil urease activity, but urease activity under K3 was lower than that under K2. Soil alkaline phosphatase activity reached its maximum under K2. This may be because potassium application stimulated soil microbial activity and increased the mineralization rate of organic phosphorus, thereby improving soil P supply. As a result, reduced P demand by the trees may have exerted an inhibitory effect on phosphatase activity (Feng, 2023). In the present study, sucrase activity increased with increasing potassium application rate, which was generally consistent with previous findings (Li, 2023). The response pattern of soil catalase activity to increasing potassium application was also consistent with the results reported by Li Jingyu (Li, 2023), who found that soil catalase activity in *Salvia miltiorrhiza* under drought stress reached its highest level under the high-potassium treatment. Nitrate reductase activity reached its maximum under K2, consistent with the findings of Zhao Tianyu’s (Zhao, 2022), who reported that increased potassium fertilization in cotton enhanced nitrate reductase activity and promoted nitrate transformation. However, nitrate reductase activity declined under K3, which may be related to changes in soil physicochemical properties and microbial community structure caused by excessive potassium application.In addition, soil enzyme activities generally decreased with increasing soil depth. This pattern may be closely associated with the enrichment of soil nutrients in the upper soil layers and the relatively greater microbial activity near the soil surface.

### 4.3 Effects of different potassium fertilization rates on root vitality of Korla fragrant pear

In this study, root vitality under all potassium treatments was higher than that under K0 across the 0–60 cm soil profile. Root vitality first increased and then decreased with increasing potassium application rate, with relatively high values observed under K2, whereas the promoting effect weakened under excessive potassium application. Fontana et al. (2020) reported that low potassium supply reduced root vitality and respiratory metabolism in plants. Potassium is involved in osmotic regulation, maintenance of ionic homeostasis, and various metabolic processes in plant cells, and an appropriate K supply is beneficial for maintaining normal root growth and metabolism (Yang et al., 2025). Xu et al. (2021) similarly found under drought conditions that an appropriate K supply promoted root growth and metabolism, whereas further increases in K supply inhibited root development, and they suggested that this shift may be related to disruption of plant ionic homeostasis caused by excessive K. This is generally consistent with the pattern observed in the present study, in which root vitality was highest under K2 but the promoting effect weakened under K3, suggesting that the physiological response of Korla fragrant pear roots to K supply may have an optimal range.

In addition, root vitality generally decreased with increasing soil depth. This pattern may be related to differences in water availability, nutrient status, and microbial activity among soil layers. Colombi et al. (2024) noted that soil physical, chemical, and biological properties exhibit pronounced spatial and temporal heterogeneity, while agricultural management practices such as fertilization and irrigation can further alter the spatial and temporal distribution of soil resources. Plant roots can, in turn, adjust their responses according to local soil conditions. Kumar et al. (2026) further pointed out that root plasticity can optimize resource acquisition under heterogeneous water conditions by regulating root depth, diameter, branching, and root hair traits. Therefore, the differences in root vitality among soil layers observed in this study may reflect physiological responses of Korla fragrant pear roots to changes in resource conditions along the soil profile. However, because water dynamics and root respiratory metabolism were not continuously monitored at different soil depths, this interpretation requires further verification.

The Mantel test showed clear depth-dependent associations between root vitality and soil nutrients and enzyme activities. The Mantel’s r values between root vitality and URE were 0.789, 0.947, and 0.940 in the 0–20, 20–40, and 40–60 cm soil layers, respectively, indicating that URE remained strongly associated with root vitality across soil depths. Root vitality was also associated to varying degrees with SOM, AK, AP, and NR. These results indicate that changes in root vitality were closely associated with soil nutrient status and enzyme activities. However, these findings represent statistical associations among variables and cannot be used to establish direct causal relationships among soil nutrients, enzyme activities, and root vitality. Overall, potassium application increased root vitality of Korla fragrant pear, but the magnitude of this effect appeared to have an appropriate range, with the promoting effect weakening under excessive potassium application. The combined patterns of soil nutrients, enzyme activities, and root vitality across soil depths indicate relatively close and depth-dependent associations among these variables, although the underlying response mechanisms remain unclear and require further investigation.

### 4.4 Effects of different potassium fertilization rates on root anatomical structure of Korla fragrant pear

Root anatomical structure provides an important structural basis for plant water and nutrient uptake, and changes in root anatomy can reflect plant adaptive strategies to external resource environments (Saengwilai et al., 2014). In this study, potassium application significantly affected the anatomical characteristics of Korla fragrant pear roots, which is consistent with the findings of Pourranjbari Saghaiesh (Pourranjbari Saghaiesh and Souri, 2018). Compared with K0, root cortex thickness generally decreased across the 0–60 cm soil profile under all potassium treatments, with relatively pronounced reductions under K2 at all soil depths. Previous studies have shown that root cortex thickness is closely related to plant nutrient supply, and that a thinner cortex and fewer cortical cell layers can reduce root construction and maintenance costs and decrease metabolic expenditure during radial transport (Chen et al., 2022; Kong et al., 2017; Lynch et al., 2021; Prince et al., 2017; Zhou et al., 2022). However, cortex function depends not only on overall thickness but also on internal structural characteristics, including cortical parenchyma cells, the proportion of aerenchyma, and the number of cell layers. Lynch et al. (2021) noted that reducing cortical burden by decreasing the number and layers of cortical parenchyma cells and increasing cortical cell size can lower root metabolic costs and improve soil resource acquisition efficiency. This is consistent with the results of the present study. Therefore, the reduction in cortex thickness following potassium application may contribute to improved nutrient use efficiency and lower metabolic costs during transport. However, because these tissue components were not further distinguished in this study, the specific mechanisms cannot yet be determined and require further verification through more detailed root histological analyses.

In this study, potassium application generally increased the proportion of cells with cellulose cell walls in the phloem of Korla fragrant pear roots, with the K2 treatment showing a relatively pronounced response across soil depths. The proportion of cells with cellulose cell walls in the xylem showed clear differences among soil depths. In the 0–40 cm soil layer, the increase was more pronounced under K2, whereas in the 40–60 cm layer, a greater increase was observed under K3. Chunyu et al. (2002) reported that potassium application can promote the synthesis of cell wall materials and improve cellular structural stability, thereby enhancing the mechanical strength and transport capacity of root tissues. Gao et al. (2019) suggested that root growth depends on the allocation of photosynthates from aboveground tissues to roots and the resulting changes in root morphology. As an important tissue for assimilate transport within plants, optimization of phloem structure may facilitate the movement of photosynthates to belowground tissues and thereby promote root growth and development. Deeken et al. (2002) reported that the AKT2/3 K^+^ channel is involved in sucrose loading into the phloem. Wu et al. (2024) further showed in navel orange that appropriate potassium application can enhance phloem loading and promote carbon flow between source and sink tissues. Tewari et al. (2021) found that insufficient potassium supply limits phloem transport, thereby affecting crop yield and quality. Therefore, the increased proportion of cells with cellulose cell walls in the phloem observed in this study may reflect an adaptive adjustment of vascular tissue structure under potassium fertilization and may be associated with changes in assimilate transport function. However, current evidence primarily demonstrates a physiological relationship between potassium and phloem loading and transport. Thus, the direct functional relationship between these anatomical changes and assimilate transport still requires further verification using indicators such as sieve-tube structure, phloem flow rate, and assimilate tracing.

In this study, root vessel area and total cross-sectional area generally increased under potassium fertilization, although the response of vessel area differed among soil depths. In the 0–20 cm soil layer, the increase was more pronounced under K2, whereas vessel area responded more strongly to K3 in the 20–40 and 40–60 cm layers, with the largest increase occurring in the 40–60 cm layer. Aritsara et al. (2023) found that larger vessel diameter was significantly associated with higher specific hydraulic conductivity. Chambi-Legoas et al. (2023) further reported that potassium fertilization enhances the plasticity of xylem traits in response to changes in water availability. Traits including vessel diameter, vessel density, vessel area, and theoretical xylem-specific hydraulic conductivity were affected by both potassium supply and water conditions and were associated with stronger post-drought growth recovery. Notably, soil nutrients and enzyme activities in the present study generally showed clear enrichment in the upper soil layers, whereas root vessel area under K3 showed the greatest increase in the 40–60 cm layer. This may be related to the vertical movement of potassium in the soil under drip irrigation and the adaptation of deeper roots to the soil water environment. HYDRUS-2D simulations by Grecco et al. (2019) showed that water movement under drip irrigation can markedly affect the spatial distribution of potassium in soil. In a fertigated vineyard in a semiarid region, Silva et al. (2018) continuously monitored soil solution at depths of 0.4 and 0.6 m and found that increasing potassium application enhanced K^+^ movement through the soil profile. Therefore, under the higher K input of the K3 treatment, part of the applied potassium may have moved to deeper soil layers with irrigation water, thereby altering the potassium supply environment of deeper roots. The large increase in root vessel area in the 40–60 cm soil layer under K3 may therefore be associated with the downward movement of potassium into deeper root zones under higher potassium input. Chambi-Legoas et al. (2023) showed that xylem traits exhibit pronounced plastic responses to potassium application rate and water conditions. On this basis, the large increase in vessel area in the 40–60 cm layer under K3 may have resulted from the combined effects of increased potassium availability in deeper soil and root water-transport requirements. However, because potassium movement across soil depths and root hydraulic traits were not continuously monitored in this study, this interpretation requires further verification.

Overall, potassium application significantly affected the anatomical structure of Korla fragrant pear roots. Zhang et al. (2021) reported that appropriate potassium fertilization can provide favorable conditions for root growth, whereas potassium deficiency or excessive potassium supply can cause nutritional imbalance and thereby inhibit plant growth. Xu et al. (2021) further indicated that potassium fertilization can have a stimulatory effect, with appropriate potassium supply improving root morphological traits and physiological functions, whereas excessive potassium supply inhibits plant growth. These findings are generally consistent with the results of the present study, indicating that appropriate potassium application can significantly optimize root structure in Korla fragrant pear. Although excessive potassium application still promoted some anatomical traits in deeper roots, the overall response was weaker than that observed under K2.

### 4.5 Effects of different potassium fertilization rates on fruit yield of Korla fragrant pear

In this study, Korla fragrant pear yield first increased and then decreased with increasing potassium application rate. Yield was highest under K2, which was 47.36% higher than that under K0, indicating that under the soil conditions, tree age, and drip-irrigation management regime of this study, appropriate potassium application was favorable for yield formation, whereas excessive potassium application did not result in any further yield increase. The PCA, Mantel test, and PLS-SEM results collectively showed continuous and close multivariate associations and structural pathways among soil physicochemical properties, enzyme activities, root traits, and yield, indicating that variation in Korla fragrant pear yield was not synchronized with any single nutrient indicator alone, but was associated with integrated changes in the belowground soil environment and root traits. This is consistent with previous studies showing that belowground ecological processes are closely related to aboveground yield performance in fruit-tree production systems (Gao et al., 2022; Liu et al., 2014).

The PCA results showed clear patterns of coordinated variation among soil nutrients, enzyme activities, root structure, and yield across different soil depths, with the first two principal components explaining more than 84% of the total variation at all three soil depths. In the 0–20 cm soil layer, SOM, AN, AP, AK, several enzyme activities, root vitality, vessel area, and cross-sectional area generally varied in the same direction along PC1, and yield also showed a positive loading on PC1, whereas pH, EC, and cortex thickness generally showed negative loadings. Prince et al. (2017) reported that increased root cortex thickness may increase radial transport resistance and metabolic costs, thereby constraining the movement of water and assimilates into the stele. These results indicate a relatively strong pattern of coordinated variation among surface-soil nutrient status, enzyme activities, root traits, and yield, whereas pH, EC, and cortex thickness showed an opposite pattern. A similar multivariate pattern was observed in the 20–40 cm soil layer, where soil nutrients, major enzyme activities, root vitality, cross-sectional area, the proportion of cells with cellulose cell walls in the phloem, and vessel area generally varied in the same direction along PC1 and shared the same loading direction as yield, whereas pH, EC, and cortex thickness were generally oriented in the opposite direction. This indicates that soil nutrients, enzyme activities, root structure, and yield remained closely associated within the 20–40 cm soil layer. In contrast, the loading patterns of some root anatomical traits changed in the 40–60 cm soil layer. In particular, the position of vessel area in the principal component space differed from that in the 0–40 cm soil layers, indicating that the responses of deeper root anatomical traits to potassium application rate and the soil environment differed from those in shallower layers. This may be because excessive vessel development and larger vessels can increase vulnerability to embolism under water deficit (Cai and Tyree, 2010; Hacke et al., 2006; Jacobsen et al., 2019), or it may simply reflect a different covariance pattern between deeper root structure and other soil and root traits. However, the loading difference itself cannot be used to determine whether vessel expansion directly promotes or suppresses yield.

The PLS-SEM model further showed significant positive path relationships between the potassium fertilization gradient and both soil physicochemical properties and root traits (β = 0.577 and 0.695, respectively, *P* < 0.001). A strong positive path relationship was also observed between soil physicochemical properties and soil enzyme activities (β = 0.944, *P* < 0.001). The path coefficient between soil enzyme activities and root traits was 0.350 (*P* = 0.034), while that between root traits and yield was 0.593 (*P* < 0.001). Within the model constructed in this study, soil physicochemical properties, enzyme activities, root traits, and yield showed a continuous structural association of “potassium fertilization gradient–soil physicochemical properties–enzyme activities–root traits–yield,” while root traits were also strongly and positively associated with yield. This was generally consistent with the multivariate association patterns revealed by PCA and the Mantel test, suggesting that roots may represent an important biological link connecting changes in the belowground soil environment with yield formation. The R^2^ value for yield in this model was 0.351, indicating that the model explained approximately 35.1% of the variation in yield and that part of the yield variation was likely associated with factors not included in the model. Yield formation in fruit trees may also be influenced by plant nutritional status, photosynthetic carbon assimilation, fruit load, water status, rhizosphere microbial communities, climatic conditions, and other factors. At the same time, the high HTMT value between Soil properties and Soil enzymes (0.993) and the relatively high SRMR values (0.222 and 0.238) indicated substantial overlap among some latent constructs, although soil nutrient status and soil enzyme activities are themselves highly coupled. In addition, separate PLS-SEM models were not constructed for the 0–20, 20–40, and 40–60 cm soil layers in this study; therefore, specific differences in structural pathways and path strengths among soil depths cannot be directly compared using the current model. Moreover, yield was used as the production-performance indicator in this experiment, whereas commercial fruit quality traits such as soluble solids, fruit firmness, aroma compounds, and peel color were not systematically measured. Therefore, the advantage of K2 was mainly reflected in its relatively favorable yield performance and integrated belowground responses under the conditions of this study.

Overall, under appropriate potassium application, Korla fragrant pear yield showed relatively consistent coordinated variation with soil nutrients, enzyme activities, and root traits, and K2 exhibited a relatively favorable integrated response under the conditions of this study. The Mantel test, PCA, and PLS-SEM consistently indicated, from different statistical perspectives, continuous and close associations among soil physicochemical properties, enzyme activities, root traits, and yield. However, these statistical relationships are not sufficient to establish a strict causal mechanism. Future studies should incorporate multi-year field experiments, continuous monitoring across multiple growth stages, plant potassium uptake and allocation, rhizosphere microbial communities, and fruit quality traits to further verify the physiological processes through which potassium fertilization influences yield formation in Korla fragrant pear.

## 5 Conclusions

Potassium fertilization significantly affected the soil–root system and yield formation of Korla fragrant pear. Across the 0–60 cm soil profile, compared with the no-potassium treatment, potassium application increased soil organic matter, alkali-hydrolyzable nitrogen, available phosphorus, and available potassium contents; enhanced the activities of urease, catalase, nitrate reductase, sucrase, and alkaline phosphatase; significantly increased root vitality; altered root structural traits; and ultimately increased Korla fragrant pear yield. Among the potassium treatments, K2 (150 kg·hm^-2^) generally showed relatively favorable performance for most soil nutrient, enzyme activity, root vitality, and yield indicators. Compared with K0, root vitality under K2 increased by 44.45%–54.19%, and yield increased by 47.36%. Further increasing the potassium application rate to K3 did not result in additional increases in root vitality or yield. The PCA, Mantel test, and PLS-SEM results collectively indicated relatively close and depth-dependent associations among soil physicochemical properties, enzyme activities, root traits, and yield. In particular, the PLS-SEM revealed a continuous structural association of “soil physicochemical properties–enzyme activities–root traits–yield.” Overall, under the soil conditions, 7–8-year-old tree age, uniform sheep manure and nitrogen and phosphorus fertilizer inputs, and drip-irrigation management regime of this study, a potassium application rate of 150 kg·hm^-2^ produced relatively favorable integrated soil–root–yield responses and may serve as a preliminary reference for potassium management of Korla fragrant pear under similar cultivation conditions. However, its long-term stability and regional applicability require further validation through multi-year, multi-site experiments conducted across multiple growth stages.

## Data Availability

The original contributions presented in the study are included in the article/**Supplementary Material**. Further inquiries can be directed to the corresponding author.
