# Supplementary material for: Effects of different potassium fertilization rates on root-soil interaction in Korla fragrant pear orchards
Source: Front Plant Sci. 2026 Oct 7;17:1899783. doi: 10.3389/fpls.2026.1899783 (PMC13683626; doi:10.3389/fpls.2026.1899783)
Supplement: Supplementary file 1 [file SupplementaryFile1.docx]

Supplementary Material

# 1 Supplementary Tables

**Table S1.** Mantel associations between root traits and soil variables at different soil depths.

| **Soil depth** | **Root trait** | **Soil Variable** | **Mantel’s r** | **P value** |
| --- | --- | --- | --- | --- |
| 0-20 cm | RV | PH | 0.397 | 0.008 |
| 0-20 cm | RV | EC | 0.620 | 0.002 |
| 0-20 cm | RV | AN | 0.506 | 0.003 |
| 0-20 cm | RV | AP | 0.339 | 0.042 |
| 0-20 cm | RV | AK | 0.661 | 0.002 |
| 0-20 cm | RV | TN | -0.101 | 0.722 |
| 0-20 cm | RV | SOM | 0.673 | 0.001 |
| 0-20 cm | RV | URE | 0.789 | 0.001 |
| 0-20 cm | RV | CAT | 0.352 | 0.022 |
| 0-20 cm | RV | SUC | 0.690 | 0.001 |
| 0-20 cm | RV | NR | 0.413 | 0.010 |
| 0-20 cm | RV | ALP | 0.413 | 0.019 |
| 0-20 cm | CT | PH | 0.303 | 0.049 |
| 0-20 cm | CT | EC | 0.595 | 0.001 |
| 0-20 cm | CT | AN | 0.577 | 0.002 |
| 0-20 cm | CT | AP | 0.182 | 0.117 |
| 0-20 cm | CT | AK | 0.600 | 0.001 |
| 0-20 cm | CT | TN | -0.146 | 0.855 |
| 0-20 cm | CT | SOM | 0.675 | 0.001 |
| 0-20 cm | CT | URE | 0.614 | 0.002 |
| 0-20 cm | CT | CAT | 0.348 | 0.025 |
| 0-20 cm | CT | SUC | 0.638 | 0.001 |
| 0-20 cm | CT | NR | 0.293 | 0.026 |
| 0-20 cm | CT | ALP | 0.318 | 0.064 |
| 0-20 cm | VA | PH | 0.208 | 0.092 |
| 0-20 cm | VA | EC | 0.546 | 0.002 |
| 0-20 cm | VA | AN | 0.799 | 0.001 |
| 0-20 cm | VA | AP | 0.499 | 0.013 |
| 0-20 cm | VA | AK | 0.424 | 0.007 |
| 0-20 cm | VA | TN | -0.092 | 0.678 |
| 0-20 cm | VA | SOM | 0.851 | 0.001 |
| 0-20 cm | VA | URE | 0.778 | 0.001 |
| 0-20 cm | VA | CAT | 0.309 | 0.030 |
| 0-20 cm | VA | SUC | 0.502 | 0.005 |
| 0-20 cm | VA | NR | 0.491 | 0.003 |
| 0-20 cm | VA | ALP | 0.529 | 0.005 |
| 0-20 cm | SA | PH | 0.625 | 0.002 |
| 0-20 cm | SA | EC | 0.835 | 0.001 |
| 0-20 cm | SA | AN | 0.438 | 0.009 |
| 0-20 cm | SA | AP | 0.069 | 0.279 |
| 0-20 cm | SA | AK | 0.842 | 0.001 |
| 0-20 cm | SA | TN | -0.146 | 0.905 |
| 0-20 cm | SA | SOM | 0.649 | 0.002 |
| 0-20 cm | SA | URE | 0.671 | 0.002 |
| 0-20 cm | SA | CAT | 0.639 | 0.001 |
| 0-20 cm | SA | SUC | 0.873 | 0.001 |
| 0-20 cm | SA | NR | 0.172 | 0.104 |
| 0-20 cm | SA | ALP | 0.236 | 0.097 |
| 20-40 cm | RV | PH | 0.361 | 0.021 |
| 20-40 cm | RV | EC | 0.737 | 0.002 |
| 20-40 cm | RV | AN | 0.563 | 0.004 |
| 20-40 cm | RV | AP | 0.853 | 0.001 |
| 20-40 cm | RV | AK | 0.786 | 0.001 |
| 20-40 cm | RV | TN | -0.028 | 0.423 |
| 20-40 cm | RV | SOM | 0.808 | 0.001 |
| 20-40 cm | RV | URE | 0.947 | 0.001 |
| 20-40 cm | RV | CAT | 0.610 | 0.003 |
| 20-40 cm | RV | SUC | 0.342 | 0.036 |
| 20-40 cm | RV | NR | 0.834 | 0.001 |
| 20-40 cm | RV | ALP | 0.434 | 0.010 |
| 20-40 cm | CT | PH | 0.373 | 0.012 |
| 20-40 cm | CT | EC | 0.686 | 0.002 |
| 20-40 cm | CT | AN | 0.475 | 0.003 |
| 20-40 cm | CT | AP | 0.765 | 0.002 |
| 20-40 cm | CT | AK | 0.797 | 0.001 |
| 20-40 cm | CT | TN | -0.029 | 0.511 |
| 20-40 cm | CT | SOM | 0.753 | 0.001 |
| 20-40 cm | CT | URE | 0.977 | 0.001 |
| 20-40 cm | CT | CAT | 0.518 | 0.004 |
| 20-40 cm | CT | SUC | 0.330 | 0.021 |
| 20-40 cm | CT | NR | 0.783 | 0.001 |
| 20-40 cm | CT | ALP | 0.450 | 0.005 |
| 20-40 cm | TA | PH | 0.239 | 0.092 |
| 20-40 cm | VA | EC | 0.384 | 0.007 |
| 20-40 cm | VA | AN | 0.333 | 0.085 |
| 20-40 cm | VA | AP | 0.416 | 0.015 |
| 20-40 cm | VA | AK | 0.468 | 0.004 |
| 20-40 cm | VA | TN | 0.095 | 0.204 |
| 20-40 cm | VA | SOM | 0.355 | 0.064 |
| 20-40 cm | VA | URE | 0.398 | 0.009 |
| 20-40 cm | VA | CAT | 0.472 | 0.006 |
| 20-40 cm | VA | SUC | 0.326 | 0.066 |
| 20-40 cm | VA | NR | 0.310 | 0.035 |
| 20-40 cm | VA | ALP | 0.111 | 0.192 |
| 20-40 cm | SA | PH | 0.508 | 0.004 |
| 20-40 cm | SA | EC | 0.809 | 0.001 |
| 20-40 cm | SA | AN | 0.459 | 0.010 |
| 20-40 cm | SA | AP | 0.829 | 0.001 |
| 20-40 cm | SA | AK | 0.794 | 0.001 |
| 20-40 cm | SA | TN | -0.135 | 0.720 |
| 20-40 cm | SA | SOM | 0.693 | 0.001 |
| 20-40 cm | SA | URE | 0.789 | 0.001 |
| 20-40 cm | SA | CAT | 0.726 | 0.001 |
| 20-40 cm | SA | SUC | 0.526 | 0.003 |
| 20-40 cm | SA | NR | 0.718 | 0.002 |
| 20-40 cm | SA | ALP | 0.438 | 0.009 |
| 40-60 cm | RV | PH | 0.348 | 0.018 |
| 40-60 cm | RV | EC | 0.854 | 0.001 |
| 40-60 cm | RV | AN | 0.346 | 0.045 |
| 40-60 cm | RV | AP | 0.301 | 0.039 |
| 40-60 cm | RV | AK | 0.790 | 0.001 |
| 40-60 cm | RV | TN | -0.094 | 0.747 |
| 40-60 cm | RV | SOM | 0.894 | 0.001 |
| 40-60 cm | RV | URE | 0.940 | 0.001 |
| 40-60 cm | RV | CAT | 0.728 | 0.001 |
| 40-60 cm | RV | SUC | 0.470 | 0.007 |
| 40-60 cm | RV | NR | 0.746 | 0.003 |
| 40-60 cm | RV | ALP | 0.486 | 0.007 |
| 40-60 cm | CT | PH | 0.671 | 0.002 |
| 40-60 cm | CT | EC | 0.691 | 0.001 |
| 40-60 cm | CT | AN | 0.377 | 0.008 |
| 40-60 cm | CT | AP | 0.488 | 0.006 |
| 40-60 cm | CT | AK | 0.817 | 0.001 |
| 40-60 cm | CT | TN | 0.037 | 0.348 |
| 40-60 cm | CT | SOM | 0.652 | 0.001 |
| 40-60 cm | CT | URE | 0.715 | 0.001 |
| 40-60 cm | CT | CAT | 0.731 | 0.001 |
| 40-60 cm | CT | SUC | 0.819 | 0.001 |
| 40-60 cm | CT | NR | 0.286 | 0.031 |
| 40-60 cm | CT | ALP | 0.602 | 0.001 |
| 40-60 cm | VA | PH | 0.915 | 0.001 |
| 40-60 cm | VA | EC | 0.867 | 0.001 |
| 40-60 cm | VA | AN | -0.006 | 0.470 |
| 40-60 cm | VA | AP | 0.113 | 0.194 |
| 40-60 cm | VA | AK | 0.914 | 0.001 |
| 40-60 cm | VA | TN | -0.102 | 0.722 |
| 40-60 cm | VA | SOM | 0.443 | 0.008 |
| 40-60 cm | VA | URE | 0.534 | 0.004 |
| 40-60 cm | VA | CAT | 0.645 | 0.001 |
| 40-60 cm | VA | SUC | 0.848 | 0.001 |
| 40-60 cm | VA | NR | 0.560 | 0.009 |
| 40-60 cm | VA | ALP | 0.099 | 0.300 |
| 40-60 cm | SA | PH | 0.516 | 0.006 |
| 40-60 cm | SA | EC | 0.917 | 0.001 |
| 40-60 cm | SA | AN | 0.299 | 0.055 |
| 40-60 cm | SA | AP | 0.346 | 0.026 |
| 40-60 cm | SA | AK | 0.876 | 0.001 |
| 40-60 cm | SA | TN | -0.070 | 0.655 |
| 40-60 cm | SA | SOM | 0.850 | 0.001 |
| 40-60 cm | SA | URE | 0.844 | 0.001 |
| 40-60 cm | SA | CAT | 0.849 | 0.001 |
| 40-60 cm | SA | SUC | 0.664 | 0.001 |
| 40-60 cm | SA | NR | 0.717 | 0.003 |
| 40-60 cm | SA | ALP | 0.438 | 0.019 |

**Table S2.** Path coefficients for the partial least squares structural equation model (PLS-SEM).

|  | **Original sample (O)** | **Sample mean (M)** | **Standard deviation (STDEV)** | **T statistics (\|O/STDEV\|)** | **P values** | **Significant or not** |
| --- | --- | --- | --- | --- | --- | --- |
| K fertilization → Root traits | 0.695 | 0.69 | 0.116 | 5.998 | 0 | Yes |
| K fertilization → Soil properties | 0.577 | 0.57 | 0.103 | 5.625 | 0 | Yes |
| Root traits → Yield | 0.593 | 0.618 | 0.071 | 8.317 | 0 | Yes |
| Soil enzymes → Root traits | 0.35 | 0.34 | 0.165 | 2.117 | 0.034 | Yes |
| Soil properties → Soil enzymes | 0.944 | 0.945 | 0.013 | 70.282 | 0 | Yes |

**Table S3.** Specific indirect effects for the partial least squares structural equation model (PLS-SEM).

|  | **Original sample (O)** | **Sample mean (M)** | **Standard deviation (STDEV)** | **T statistics (\|O/STDEV\|)** | **P values** | **Significant or not** |
| --- | --- | --- | --- | --- | --- | --- |
| Soil properties → Soil enzymes → Root traits | 0.33 | 0.322 | 0.156 | 2.117 | 0.034 | Yes |
| K fertilization → Root traits → Yield | 0.412 | 0.425 | 0.082 | 4.997 | 0 | Yes |
| Soil enzymes → Root traits → Yield | 0.207 | 0.211 | 0.105 | 1.972 | 0.049 | Yes |
| K fertilization → Soil properties → Soil enzymes → Root traits → Yield | 0.113 | 0.118 | 0.062 | 1.81 | 0.07 | Not |
| Soil properties → Soil enzymes → Root traits → Yield | 0.196 | 0.2 | 0.099 | 1.969 | 0.049 | Yes |
| K fertilization → Soil properties → Soil enzymes → Root traits | 0.191 | 0.189 | 0.097 | 1.967 | 0.049 | Yes |
| K fertilization → Soil properties → Soil enzymes | 0.545 | 0.539 | 0.101 | 5.378 | 0 | Yes |

**Table S4.** Heterotrait-monotrait ratio (HTMT) for the partial least squares structural equation model (PLS-SEM).

|  | **Original sample (O)** | **Sample mean (M)** | **0.025** | **0.975** |
| --- | --- | --- | --- | --- |
| Root traits ↔ K fertilization | 0.944 | 0.942 | 0.814 | 1.045 |
| Soil enzymes ↔ K fertilization | 0.356 | 0.354 | 0.092 | 0.607 |
| Soil enzymes ↔ Root traits | 0.783 | 0.789 | 0.629 | 0.949 |
| Soil properties ↔ K fertilization | 0.565 | 0.579 | 0.452 | 0.733 |
| Soil properties ↔ Root traits | 0.832 | 0.848 | 0.712 | 0.99 |
| Soil properties ↔ Soil enzymes | 0.993 | 0.993 | 0.973 | 1.008 |
| Yield ↔ K fertilization | 0.567 | 0.57 | 0.362 | 0.757 |
| Yield ↔ Root traits | 0.68 | 0.712 | 0.504 | 0.921 |
| Yield ↔ Soil enzymes | 0.275 | 0.336 | 0.179 | 0.525 |
| Yield ↔ Soil properties | 0.345 | 0.391 | 0.258 | 0.554 |

**Table S5.** Average variance extracted (AVE) for the partial least squares structural equation model (PLS-SEM).

|  | **Original sample (O)** | **Sample mean (M)** | **Standard deviation (STDEV)** | **T statistics (\|O/STDEV\|)** | **P values** |
| --- | --- | --- | --- | --- | --- |
| K fertilization | 1 | 1 | 0 | n/a | n/a |
| Root traits | 0.51 | 0.51 | 0.054 | 9.432 | 0 |
| Soil enzymes | 0.891 | 0.89 | 0.021 | 41.717 | 0 |
| Soil properties | 0.813 | 0.811 | 0.042 | 19.33 | 0 |
| Yield | 0.716 | 0.702 | 0.083 | 8.614 | 0 |

**Table S6.** Composite reliability (rho_c) for the partial least squares structural equation model (PLS-SEM).

|  | **Original sample (O)** | **Sample mean (M)** | **Standard deviation (STDEV)** | **T statistics (\|O/STDEV\|)** | **P values** |
| --- | --- | --- | --- | --- | --- |
| K fertilization | 1 | 1 | 0 | n/a | n/a |
| Root traits | 0.798 | 0.788 | 0.064 | 12.442 | 0 |
| Soil enzymes | 0.97 | 0.97 | 0.006 | 150.597 | 0 |
| Soil properties | 0.751 | 0.752 | 0.047 | 16.107 | 0 |
| Yield | 0.882 | 0.868 | 0.07 | 12.688 | 0 |

**Table S7.** Standardized root mean square residual (SRMR) for the partial least squares structural equation model (PLS-SEM).

|  | **Original sample (O)** | **Sample mean (M)** | **0.95** | **0.99** |
| --- | --- | --- | --- | --- |
| Saturated model | 0.222 | 0.075 | 0.099 | 0.113 |
| Estimated model | 0.238 | 0.085 | 0.118 | 0.137 |

**Table S8.** Inner variance inflation factor (VIF) values for the partial least squares structural equation model (PLS-SEM).

|  | **VIF** |
| --- | --- |
| K fertilization → Root traits | 1.14 |
| K fertilization → Soil properties | 1 |
| Root traits → Yield | 1 |
| Soil enzymes → Root traits | 1.14 |
| Soil properties → Soil enzymes | 1 |

**Table S9.** Outer loadings for the partial least squares structural equation model (PLS-SEM).

|  | **Original sample (O)** | **Sample mean (M)** | **Standard deviation (STDEV)** | **T statistics (\|O/STDEV\|)** | **P values** |
| --- | --- | --- | --- | --- | --- |
| AK ← Soil properties | 0.96 | 0.959 | 0.013 | 74.158 | 0 |
| ALP ← Soil enzymes | 0.963 | 0.964 | 0.008 | 117.156 | 0 |
| AN ← Soil properties | 0.899 | 0.899 | 0.027 | 33.55 | 0 |
| AP ← Soil properties | 0.823 | 0.822 | 0.046 | 18.032 | 0 |
| CAT ← Soil enzymes | 0.94 | 0.94 | 0.015 | 61.373 | 0 |
| EC ← Soil properties | -0.895 | -0.889 | 0.038 | 23.636 | 0 |
| Fertilization methods ← K fertilization | 1 | 1 | 0 | n/a | n/a |
| NR ← Soil enzymes | 0.904 | 0.901 | 0.03 | 30.534 | 0 |
| PCCWCP ← Root traits | 0.898 | 0.886 | 0.065 | 13.792 | 0 |
| PCCWCX ← Root traits | 0.673 | 0.655 | 0.124 | 5.43 | 0 |
| Rv ← Root traits | 0.756 | 0.74 | 0.139 | 5.443 | 0 |
| SFA ← Yield | 0.758 | 0.731 | 0.158 | 4.802 | 0 |
| SFM ← Yield | 0.852 | 0.851 | 0.075 | 11.382 | 0 |
| SOM ← Soil properties | 0.94 | 0.94 | 0.018 | 52.835 | 0 |
| URE ← Soil enzymes | 0.968 | 0.967 | 0.011 | 91.969 | 0 |
| VA ← Root traits | 0.455 | 0.46 | 0.164 | 2.775 | 0.006 |
| Yd ← Yield | 0.921 | 0.897 | 0.119 | 7.726 | 0 |
| pH ← Soil properties | -0.887 | -0.882 | 0.042 | 21.262 | 0 |
